# Increases in Northern Hemisphere winter circulation waviness over 1989 to 2011 driven by decadal oceanic variability

**DOI:** 10.1126/sciadv.aeg2637

**Published:** 2026-11-13

**Authors:** Weiming Ma, Hailong Wang, Sandro W. Lubis, Gang Chen

**Affiliations:** ^1^ Multi-Scale & Sector Dynamics Division, Pacific Northwest National Laboratory, Richland, WA, USA.; ^2^ Department of Atmospheric and Oceanic Sciences, University of California, Los Angeles, CA, USA.

## Abstract

Large-scale circulation waviness shapes day-to-day weather and extremes. During 1989 to 2011, Northern Hemisphere midlatitudes experienced a pronounced increase in wintertime waviness, coinciding with rapid Arctic warming and more frequent cold-air outbreaks. While Arctic amplification was hypothesized to cause this trend via jet weakening, subsequent studies have failed to support this mechanism, leaving the origin unresolved. Here, using large-ensemble simulations, statistical analyses, and dynamical diagnostics, we demonstrate that variability in sea surface temperature and sea ice accounts for approximately half (48.7 to 63.3%) of the observed increase. A positive phase shift of the Atlantic Multidecadal Oscillation (AMO) is the prominent driver, enhancing waviness across northern Eurasia, the northwest Pacific, and the North Atlantic. A concurrent negative Interdecadal Pacific Oscillation shift partially offsets the AMO-driven anomalies in the northwest Pacific. Projections indicate that internal variability will outweigh model differences as the main source of near-term uncertainty. These results refine attribution of recent winter waviness changes and identify an AMO-linked source of predictability for long-term shifts in midlatitude extremes.

## INTRODUCTION

Recent decades have witnessed more rapid Arctic warming than global mean, commonly referred to as Arctic amplification (AA) (*1*). Coinciding with AA, planetary-scale waves across the Northern Hemisphere exhibited increased waviness during the cold season over a limited period, particularly between the early 1990s and around 2010 (*2*–*5*). This short-term atmospheric circulation change, together with AA, coincided with wintertime cooling over midlatitude Eurasia (*6*), often referred to as the “warm Arctic, cold continents” pattern, raising the debate about whether the amplified Arctic warming and the enhanced waviness are causally connected. A central and controversial idea was proposed by Francis and Vavrus (*2*), who hypothesized that AA weakens the equator-to-pole temperature gradient, slows the westerly jet, and thereby enhances wave amplitude, leading to a wavier midlatitude circulation. This framework implies that the observed short-term increase in waviness may have been primarily driven by anthropogenic forcing, as the recent historical AA has been largely attributed to externally forced temperature changes, although internal variability was identified to play a secondary role (*7*, *8*).

Since its publication, the Francis and Vavrus (*2*) hypothesis has attracted substantial attention. On one hand, it resonated strongly with the public and was frequently cited by the media as an explanation for recent weather extremes, reinforcing the perception that continued Arctic warming will amplify midlatitude extremes (*9*–*11*). On the other hand, the hypothesis ignited vigorous debate within the scientific community and stimulated a proliferation of studies on Arctic-midlatitude linkages (*12*–*15*). Observational analyses have shown that apparent trends in the circulation waviness are sensitive to the selected time period and to the choice of diagnostic metrics (*3*, *4*, *16*–*19*). Modeling experiments with prescribed Arctic sea-ice loss, intended to isolate the influence of AA, generally produce very weak or absent responses in waviness (*4*, *20*–*24*), which are often overwhelmed by internal variability, and in some cases even suggest a reduction in waviness (*25*). Moreover, despite the continued AA, model projections indicate a weak decline in waviness under future warming (*19*, *26*–*28*). Together, these findings have led many studies to conclude that the observed short-term increase in Northern Hemisphere waviness from the early 1990s to around 2010 is unlikely to be driven by AA, and thus lacks a robust anthropogenic origin, but is instead more consistent with internal variability.

If the observed short-term increase in waviness cannot be explained by AA, what then drives the trend? Here, using multimodel ensemble simulations, statistical analyses, and a dynamical theory based on the finite-amplitude local wave activity (LWA) (*29*), we show that the enhanced waviness trend during 1989 to 2011 is unlikely to have arisen from anthropogenic forcing, consistent with previous studies. Instead, it reflects internal variability, with an important and substantial role played by the negative-to-positive phase transition of the Atlantic Multidecadal Oscillation (AMO). This mechanism highlights the partially predictable nature of the observed decadal waviness variability. In future scenarios, models project only modest changes in waviness under the continued warming, with the magnitude and sign of the response varying across models, where uncertainty is dominated by internal variability rather than structural model differences. By identifying the AMO as a substantial source of internal variability, our results provide a pathway to reducing uncertainty in the response of circulation waviness and, thereby, in midlatitude extreme weather, under future warming.

## RESULTS

### Observed and simulated trends in LWA

Motivated by Blackport and Screen (*4*), we quantify circulation waviness using LWA, an objective metric based on the meridional displacement of 500-hPa geopotential height contours [(*30**,* *31*); Materials and Methods]. Blackport and Screen (*4*) used LWA to show that the late 20th-century to early 21st-century Northern Hemisphere winter waviness trend is strongly period dependent, and AA alone cannot produce an increase in waviness. Using the same waviness metric here enables a direct comparison with their results and provides a consistent framework to further diagnose the dominant driver of the observed trend. Consistent with Blackport and Screen (*4*) (their figure S3), the Northern Hemisphere midlatitudes experienced enhanced waviness during the cold season (December to February) from 1989 to 2011 (Fig. 1, A and E). The most pronounced increases occurred over the North Atlantic poleward of ∼40°N and northern Eurasia, with particularly strong trends across northeastern Eurasia. At the same time, notable negative LWA trends were largely confined to lower latitudes over the North Atlantic (∼30°N), extending eastward into western Europe. The spatial pattern and magnitude of the European Centre for Medium-Range Weather Forecasts Reanalysis, version 5 (ERA5) trends shown in Fig. 1A are corroborated by other reanalyses (fig. S1), underscoring the robustness of the observed increase in waviness.

**Fig. 1.**
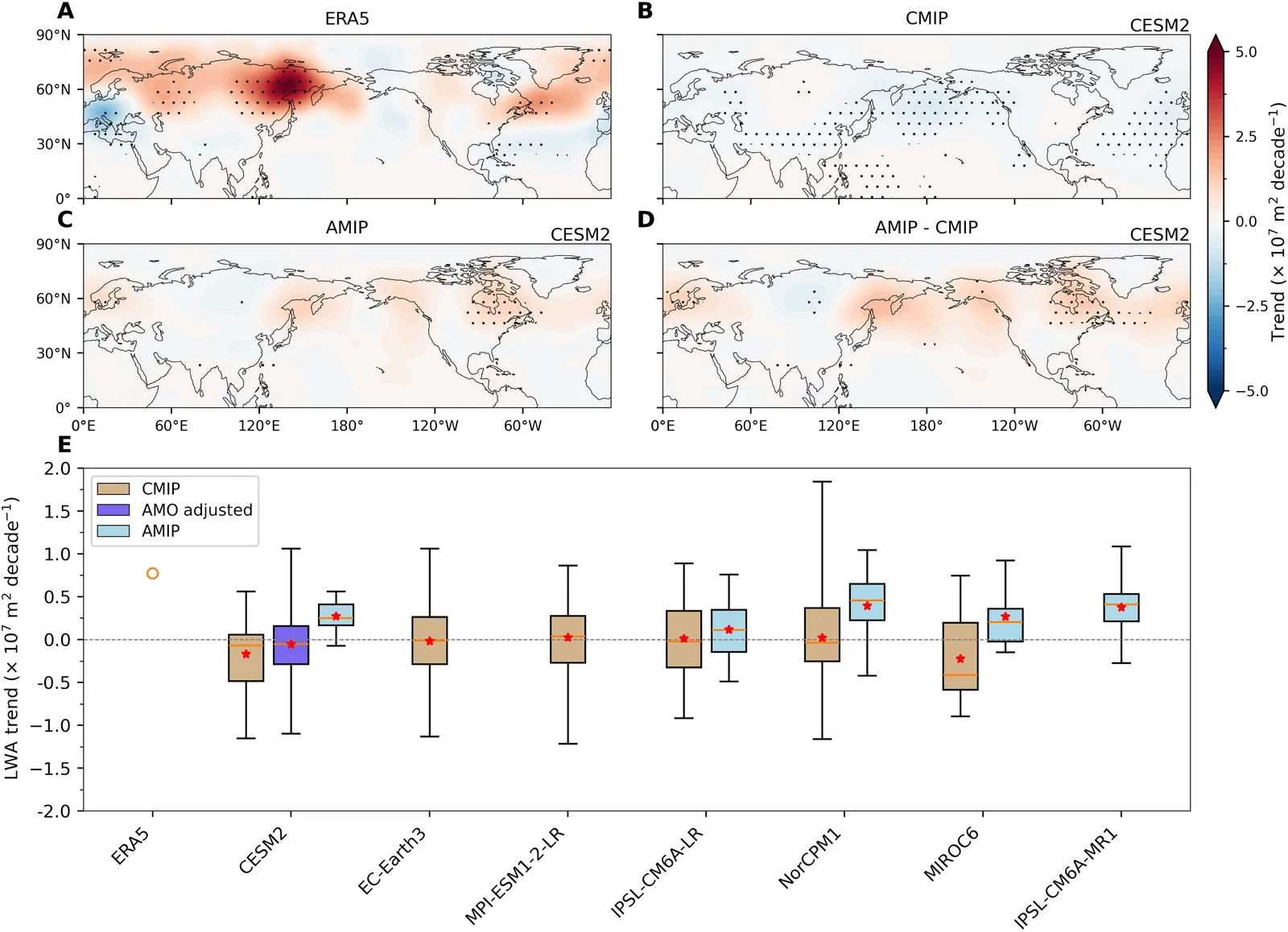
The cold season LWA trends from 1989 to 2011 in ERA5 and ensemble simulations. (**A**) LWA trends in ERA5. (**B**) Ensemble mean LWA trends in CESM2 Large Ensemble. (**C**) Ensemble mean LWA trends in CESM2 AMIP ensemble. (**D**) Difference in the ensemble mean LWA trends between CESM2 AMIP ensemble and CESM2 Large Ensemble. (**E**) Average LWA trends over the Northern Hemisphere midlatitudes (40°N to 80°N) in ERA5 (circle) and ensemble simulations (bars and whiskers). The orange lines (red stars) in (E) represent the ensemble median (mean). The boxes represent the 25th to 75th percentile range of the spread. The whiskers denote the maximum and minimum of the spread. The stippled areas in (A), (B), (C), and (D) indicate that trends are significant at the 0.05 level based on the Student’s *t* test.

To investigate driving mechanisms of the enhanced LWA trend, we first analyze ensemble-mean LWA trends from five single-model large ensembles selected based on the availability of daily 500-hPa geopotential height, sufficiently large historical ensemble sizes, and, for consistency with our future projection analyses, corresponding SSP370 simulations. The CESM2 Large Ensemble and four additional ensembles (Fig. 1B and fig. S2) indicate that anthropogenic forcing alone produces only a weak response in LWA trends, with the magnitude, sign, and spatial pattern varying across models. Notably, CESM2 Large Ensemble even simulates a slight decline in midlatitude waviness. Moreover, no individual CESM2 Large Ensemble member reproduces a trend as strong as the observed one (Fig. 1E). Examination of the other large ensembles shows that the observed trend also lies near the upper tail of their simulated distributions, although it is not entirely outside the range of all models: only 3, 2, 2, and 3 members exceed the observed trend in the 50-member EC-Earth3 ensemble, 50-member MPI-ESM1-2-LR ensemble, 33-member IPSL-CM6A-LR ensemble, and 30-member NorCPM1 ensemble, respectively. These results underscore the extreme nature of the observed increase and suggest that models may underestimate the amplitude of the relevant decadal-to-multidecadal LWA variability, or that the observed trend represents a rare realization of internal variability (Fig. 1E). Consistent with earlier studies (*4*, *19*, *21*), these results demonstrate that the recent LWA trend cannot be attributed to anthropogenic forcing, and instead point to the critical role of internal variability.

Internal variability can broadly be decomposed into two components: (i) a less predictable component originating from the atmosphere, and (ii) a more predictable component associated with boundary conditions, particularly the ocean surface, including both sea surface temperature (SST) and sea-ice concentration. To assess whether ocean-driven internal variability contributed to the enhanced LWA trend, we analyze a 10-member Atmospheric Model Intercomparison Project (AMIP)–type ensemble based on CESM2, forced with observed SST and sea-ice variability. During the study period, the prescribed SST trends are characterized by strong and statistically significant warming over the North Atlantic, together with a cooling signal over the tropical eastern Pacific that extends poleward along the eastern Pacific basin (fig. S3). Compared with fully coupled CESM2 Large Ensemble, CESM2 AMIP ensemble produces substantially stronger LWA trends across the Northern Hemisphere (Fig. 1, C to E), despite simulating a spurious weak negative trend over central northern Eurasia and a positive trend over northwestern North America. Notably, CESM2 AMIP ensemble reproduces the enhanced waviness over the northern North Atlantic and captures the strengthened LWA trend over northeastern Eurasia, albeit slightly displaced eastward. This behavior is not unique to CESM2. Results from four additional AMIP ensembles with at least 10 members (fig. S4) show a broadly consistent picture: except for IPSL-CM6A-LR, the ensembles from MIROC6, NorCPM1, and IPSL-CM6A-MR1 all simulate positive LWA trends across the Northern Hemisphere, with enhanced activity over the northern North Atlantic and northern Eurasia, thereby capturing several key regional features of the observed pattern, although the detailed spatial alignment with observations remains imperfect. Moreover, in models where both AMIP and CMIP ensembles are available, the AMIP simulations (again except for IPSL-CM6A-LR) consistently yield stronger positive LWA trends than their CMIP counterparts (fig. S4 and Fig. 1E).

We further quantify this ocean-forced contribution by comparing the ensemble-mean LWA trends between paired AMIP and coupled simulations from the same model. Because these simulations are subject to the same external forcing, their ensemble-mean difference primarily reflects the influence of observed SST and sea-ice variability. Averaged over the Northern Hemisphere midlatitudes (40° to 80°N), the observed LWA trend is approximately 7.71 × 10^6^ m^2^ decade^−1^. For CESM2, MIROC6, NorCPM1, and IPSL-CM6A-LR, the AMIP-coupled differences are 4.42 × 10^6^, 4.88 × 10^6^, 3.76 × 10^6^, and 1.06 × 10^6^ m^2^ decade^−1^, respectively, corresponding to 57.3, 63.3, 48.7, and 13.8% of the observed trend (fig. S5). The IPSL-CM6A-LR AMIP ensemble does not reproduce a statistically significant positive ensemble-mean LWA trend, and the observed trend falls outside the spread of its 22 members (Fig. 1E); excluding this model yields a multimodel mean contribution of 56.4% across CESM2, MIROC6, and NorCPM1. The observed trend also falls outside the CESM2 AMIP ensemble spread (Fig. 1E). We therefore additionally calculate the contribution using only MIROC6 and NorCPM1, which yields a nearly identical estimate of 56.0%. Thus, regardless of whether CESM2 is included, observed SST and sea-ice variability accounts for approximately half of the observed waviness increase. For comparison, including IPSL-CM6A-LR yields a four-model mean contribution of 45.8%, indicating that even under this more inclusive model selection, observed ocean boundary variability accounts for nearly half of the observed increase. Given the commonly recognized weak signal-to-noise ratio in model responses to multidecadal SST variability (*32*–*34*), the 56.0 to 56.4% estimates are likely conservative. Together, these results provide quantitative evidence that observed ocean boundary variability was capable of producing an LWA response that shares key regional features with the observed trend and made a substantial contribution to the recent enhancement of circulation waviness over the Northern Hemisphere midlatitudes.

### The prominent role of AMO in driving the observed LWA trends

To identify modes of oceanic internal variability associated with the enhanced LWA trend, we apply maximum covariance analysis (MCA) to the covariance between internally generated midlatitude LWA trends (40° to 80°N) and global SST trends (60°S to 70°N) in five large ensembles (CESM2 Large Ensemble, EC-Earth3, MPI-ESM1-2-LR, IPSL-CM6A-LR, and NorCPM1; each ≥30 members). For each ensemble, we construct a pseudo-ensemble (*35*, *36*) of 23-year trends by dividing the historical simulation of each member into consecutive 23-year segments and computing segment-wise LWA and SST trends. We then remove the corresponding ensemble-mean trends to isolate the internally generated components. Because ensemble sizes and historical-period lengths differ across models (Materials and Methods), this procedure yields pseudo-ensembles of 7100 members for CESM2 Large Ensemble, 1100 for EC-Earth3, 7100 for MPI-ESM1-2-LR, 1386 for IPSL-CM6A-LR, and 1260 for NorCPM1. MCA is then applied to each pseudo-ensemble separately.

The multiensemble-mean leading MCA mode features widespread positive LWA trends across the Northern Hemisphere, with the strongest signal over the northern North Pacific (Fig. 2A). The associated SST pattern resembles a positive Interdecadal Pacific Oscillation (IPO)–like configuration over the Pacific (Fig. 2B and fig. S6B). Over the Atlantic, SST trends are predominantly positive north of ∼30°S, with weaker and insignificant cooling trends over parts of the western midlatitude North Atlantic, broadly consistent with a positive AMO-like structure (fig. S6A). As shown previously, a concurrent positive phase shift of the IPO and AMO can amplify Arctic warming (*37*). Thus, this leading MCA mode indicates that AA and enhanced midlatitude waviness can co-occur as parallel responses to ocean-driven internal variability, without requiring a causal link between them. In contrast, the second MCA mode combines a negative IPO-like Pacific pattern with more pronounced subpolar North Atlantic warming, a hallmark of the positive phase of the AMO (Fig. 2D; cf. fig. S6A). Notably, this coupled SST trend pattern mirrors the observed late 20th-century to early 21st-century evolution, characterized by a negative IPO phase shift alongside a positive AMO phase shift (fig. S3). The corresponding LWA trend pattern closely reproduces the observed spatial structure (Fig. 2C). Across the five ensembles, these two modes are generally robust (figs. S7 and S8), with modest intermodel differences in the detailed Atlantic SST expression; in NorCPM1, the pattern corresponding to this “second-mode” structure appears as MCA mode 3. Together, these results indicate that the observed LWA trend is most strongly associated with the combination of a negative IPO-like Pacific state and AMO-like North Atlantic warming, rather than either basin acting in isolation.

**Fig. 2.**
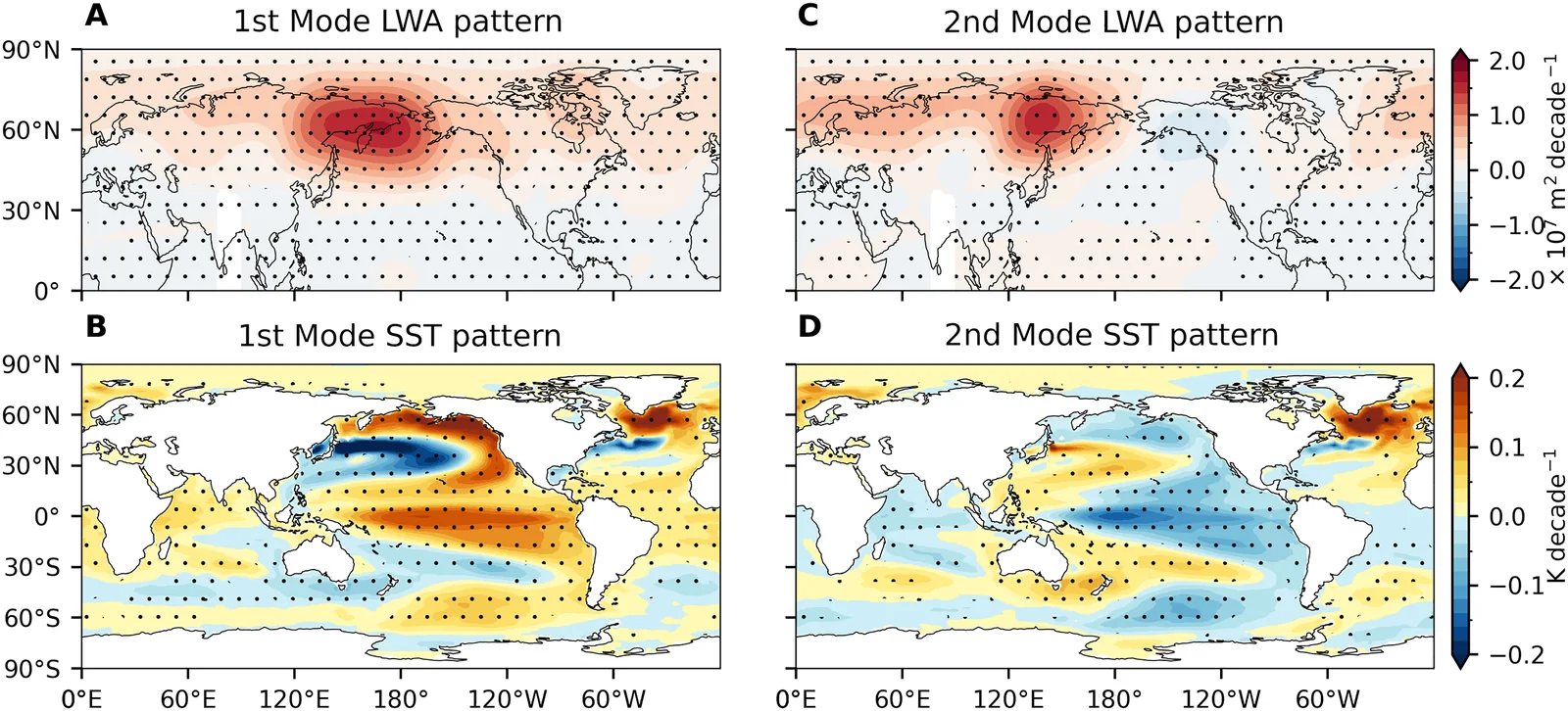
Internally generated covarying patterns of LWA trends and sea surface temperature (SST) trends. (**A**) Multiensemble-mean LWA trend pattern associated with the first mode of maximum covariance analysis (MCA). (**B**) Multiensemble-mean SST trend pattern associated with the first mode of MCA. (**C** and **D**) Same as (A) and (B), but for the second mode of MCA. For each ensemble, internally generated trends are first obtained by removing the ensemble-mean trend from each member. The resulting member-to-member trend anomalies are then regressed onto the standardized MCA expansion coefficients, and the regression patterns are lastly averaged across the five ensembles (CESM2 Large Ensemble, EC-Earth3, MPI-ESM1-2-LR, IPSL-CM6A-LR, and NorCPM1). Stippling denotes grid points where at least four of five ensembles agree on the sign of the multiensemble-mean pattern.

To further assess these connections, we apply a fingerprint-based analysis following Sun *et al.* (*38*). For each model pseudo-ensemble, we calculate the spatial correlation between the observed LWA trend pattern (Fig. 1A) and each internally generated 23-year LWA trend over the Northern Hemisphere midlatitudes (40° to 80°N). Members with correlations ≥0.5 and ≤−0.5 are selected as positive- and negative-correlation members, respectively. The difference in ensemble-mean LWA trends between these two groups closely reproduces the observed spatial pattern (fig. S9A). The associated SST trend difference shows a negative IPO-like pattern over the Pacific together with a positive AMO-like pattern over the North Atlantic (fig. S9B). Similar LWA and SST trend patterns can also be identified in individual models (fig. S10), although the negative IPO-like structure is less coherent in IPSL-CM6A-LR and NorCPM1, and the positive AMO-like warming in NorCPM1 is more confined to higher latitudes. This reduced coherence is likely related to sampling: on average, only about 17% of pseudo-ensemble members are retained in this fingerprint analysis, whereas the MCA uses all members and therefore yields more robust covariance patterns. As a result, atmospheric internal variability and other sources of noise have a larger influence on the fingerprint composites. Nevertheless, these results further corroborate the link between the observed LWA trend pattern and a combined negative IPO-like and positive AMO-like SST trend pattern.

Last, we assess the relative roles of the IPO and AMO in the observed LWA trend. For each ensemble member, we regress LWA variability onto that member’s unstandardized AMO index, yielding an AMO-LWA sensitivity in units of square meters per kelvin; these member-specific regression coefficients are then averaged within each model ensemble and across models. The resulting multiensemble-mean LWA regression pattern associated with a positive AMO closely matches the observed spatial structure. It features pronounced waviness increases over northern Eurasia and the North Atlantic sector, with the main discrepancy being that the strengthened signal over northeastern Eurasia extends somewhat farther eastward than observed (Fig. 3A). To quantify the magnitude of this AMO-associated response, we compare it with the observed LWA trend normalized by the observed AMO trend. During 1989 to 2011, the observed LWA trend averaged over the Northern Hemisphere midlatitudes (40° to 80°N) is approximately 7.71 × 10^6^ m^2^ decade^−1^, while the observed AMO trend is approximately 0.236 K decade^−1^, corresponding to 3.27 × 10^7^ m^2^ K^−1^. The member-specific AMO-LWA sensitivities exhibit substantial intermember spread within each ensemble, with most members producing a positive response but some producing negative values, highlighting the modulation of the AMO-associated response by internal variability (fig. S11A). The ensemble-mean sensitivities range from 3.86 × 10^6^ to 9.16 × 10^6^ m^2^ K^−1^ across the five large ensembles, accounting for 11.8 to 28.1% of the observed normalized LWA trend, with a multimodel mean of 19.2% (fig. S11B). These percentages should be interpreted as an approximate measure of the magnitude of the model-simulated linear AMO-associated LWA response relative to the observed AMO-normalized trend, rather than as a direct attribution of a specific fraction of the observed LWA trend to the AMO. The observed normalized value implicitly assumes a linear relationship between the observed LWA and AMO changes, while the model-derived sensitivities represent only the average linear relationship sampled within the individual simulations; other modes of variability and externally forced changes can also contribute to the observed and simulated LWA variations. Moreover, the observed positive AMO phase shift had its own specific spatial pattern and temporal evolution and occurred concurrently with a negative IPO phase shift—a relatively rare combination in the model world, as indicated by the second MCA mode (fig. S8). The associated changes in the background large-scale circulation may interact with the AMO-related forcing, thereby modulating, or potentially amplifying, the influence of the AMO on LWA variability.

**Fig. 3.**
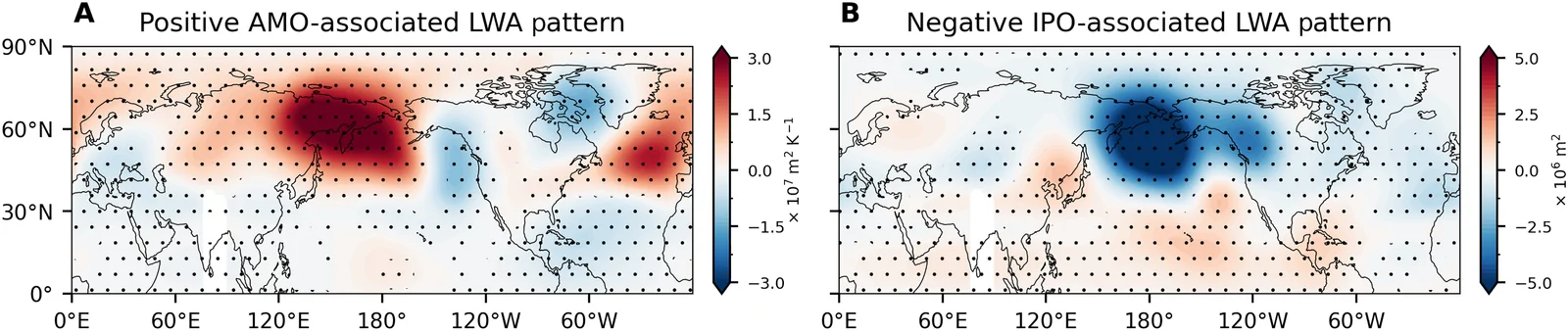
LWA pattern associated with Atlantic Multidecadal Oscillation (AMO) and Interdecadal Pacific Oscillation (IPO). (**A**) Multiensemble-mean LWA regression pattern associated with positive AMO, obtained by regressing LWA variability onto the unstandardized AMO index. (**B**) Multiensemble-mean LWA regression pattern associated with negative IPO, obtained by regressing LWA variability onto the standardized IPO index and reversing the sign so that the pattern corresponds to the negative IPO phase. Multiensemble-mean patterns are computed by averaging the ensemble-mean regression patterns from five large ensembles (CESM2 Large Ensemble, EC-Earth3, MPI-ESM1-2-LR, IPSL-CM6A-LR, and NorCPM1). Stippling denotes grid points where at least four of five ensembles agree on the sign of the multiensemble-mean pattern.

Consistent with the important role of the AMO, statistically adjusting the AMO evolution in each CESM2 Large Ensemble member to follow the observed AMO trajectory (Materials and Methods) shifts the distribution of simulated LWA trends upward, with three members producing trends that exceed the observed magnitude (Fig. 1E). In contrast, the LWA pattern associated with a negative IPO is characterized by strong negative anomalies over the North Pacific and northwestern North America, along with a weaker but still significant decrease over parts of the northern North Atlantic (Fig. 3B). Because the IPO index is defined as the principal component of the leading empirical orthogonal function (EOF) mode of detrended Pacific SST anomalies, the SST changes associated with a unit change in the IPO index are not directly comparable between models and observations. Therefore, the IPO regression patterns cannot be used to provide a direct quantitative estimate of the observed IPO contribution. Nevertheless, averaged over the Northern Hemisphere midlatitudes, the LWA anomalies associated with the negative IPO are negative in all models, indicating that the negative IPO phase shift tends, on average, to suppress LWA.

The spatial pattern further supports this interpretation. The North Pacific weakening associated with a negative IPO counteracts the AMO-related positive anomaly there, helping to confine the observed positive LWA trend primarily to northeastern Eurasia (Fig. 1A). Over Eurasia, the IPO-related LWA signal is comparatively small in the multiensemble mean because its sign is model dependent, positive in CESM2 Large Ensemble and MPI-ESM1-2-LR but negative in IPSL-CM6A-LR and NorCPM1 (fig. S12), yielding an overall weak contribution in Fig. 3B. Over North America, the positive AMO- and negative IPO-associated patterns tend to oppose each other and partially cancel, consistent with the muted observed trend in that region (Fig. 1A).

The AMO and IPO regression patterns also help clarify the interpretation of the MCA modes and address the concern that, if the first MCA mode primarily reflects model bias or a model-preferred variability pattern that does not exist in reality, it could bias the inferred LWA regression patterns associated with the AMO or IPO. Because MCA modes are ranked by the amount of SST-LWA covariance explained within the model pseudo-ensemble, the fact that the second MCA mode more closely resembles the observed 1989 to 2011 event does not necessarily imply that the first MCA mode is spurious. The physically defined AMO and IPO regression patterns provide a consistent interpretation of the MCA results: the AMO-related pattern features enhanced LWA over northern Eurasia and the North Atlantic sector, whereas the IPO-related pattern captures strong LWA variability over the northern North Pacific and North American sectors. These patterns qualitatively explain the main features of both MCA modes, suggesting that the first MCA mode does not merely represent an arbitrary model-preferred mode or a spurious model bias. Rather, it appears to reflect a physically interpretable mode of coupled SST-LWA variability associated with AMO- and IPO-related variability, even though the observed 1989 to 2011 event projects more strongly onto the second MCA mode.

Together, these results indicate that the positive AMO makes an important and substantial contribution to the observed LWA trend, while the negative IPO plays a secondary, modulatory role. Although the available datasets do not allow us to provide a clean quantitative separation of the observed AMO and IPO contributions, the combined evidence supports the interpretation that the 56.0 to 56.4% net SST-driven contribution estimated from the AMIP-CMIP differences can be viewed as a conservative lower bound for the contribution of the observed positive AMO phase shift. This is because the observed SST-driven contribution is positive and explains approximately half of the observed LWA increase, whereas the negative IPO phase shift acts in the opposite direction by suppressing hemispheric-mean LWA. Therefore, the positive AMO phase shift must not only contribute to the positive SST-driven LWA trend, but also partly offset the damping effect associated with the negative IPO. In this sense, the actual contribution of the observed AMO phase shift is likely larger than the net SST-driven contribution estimated from the AMIP-CMIP differences. We therefore conclude that the observed positive AMO phase shift made a substantial contribution to the observed LWA increase and played a prominent role in shaping its spatial structure, while the negative IPO phase shift mainly damped the trend over the North Pacific and parts of the northern North Atlantic and exerted a more uncertain influence over Eurasia.

### Mechanisms of the LWA trends

Thus far, we have shown, using multiple coupled-model and atmosphere-only large ensembles, that observed SST and sea-ice variability is closely associated with and capable of contributing to the observed LWA trends (Fig. 1 and figs. S2 and S4). Using complementary statistical analyses, we further identify a prominent role for IPO/AMO phase shifts. To further understand this link, we now turn to a dynamical framework. The LWA metric used thus far has been based on 500-hPa geopotential height, chosen for its simplicity and computational efficiency and to enable a direct comparison with Blackport and Screen (*4*). However, this metric lacks a firm dynamical foundation, limiting mechanistic insight. To address this limitation, we adopt the LWA framework formulated in terms of quasi-geostrophic potential vorticity (QGPV) (*29*). This formulation benefits from a prognostic equation that explicitly relates QGPV LWA tendencies to individual dynamical processes (Materials and Methods), thereby enabling a more process-based understanding of the observed LWA trends. As shown in Fig. 4A, the observed QGPV-based LWA trend pattern closely matches that derived from 500-hPa geopotential height (Fig. 1A), except for a somewhat weaker signal over northeastern Eurasia. Across the pseudo-ensemble members in CESM2 Large Ensemble, the two formulations yield highly consistent magnitudes and spatial correlations (fig. S13). Likewise, the MCA results are insensitive to whether LWA is defined using QGPV or 500-hPa height (fig. S14 versus figs. S7, A and B, and S8, A and B). These findings confirm that our main conclusions are robust as to the choice of LWA metric and thus justify the use of 500-hPa geopotential height-based LWA in this study.

**Fig. 4.**
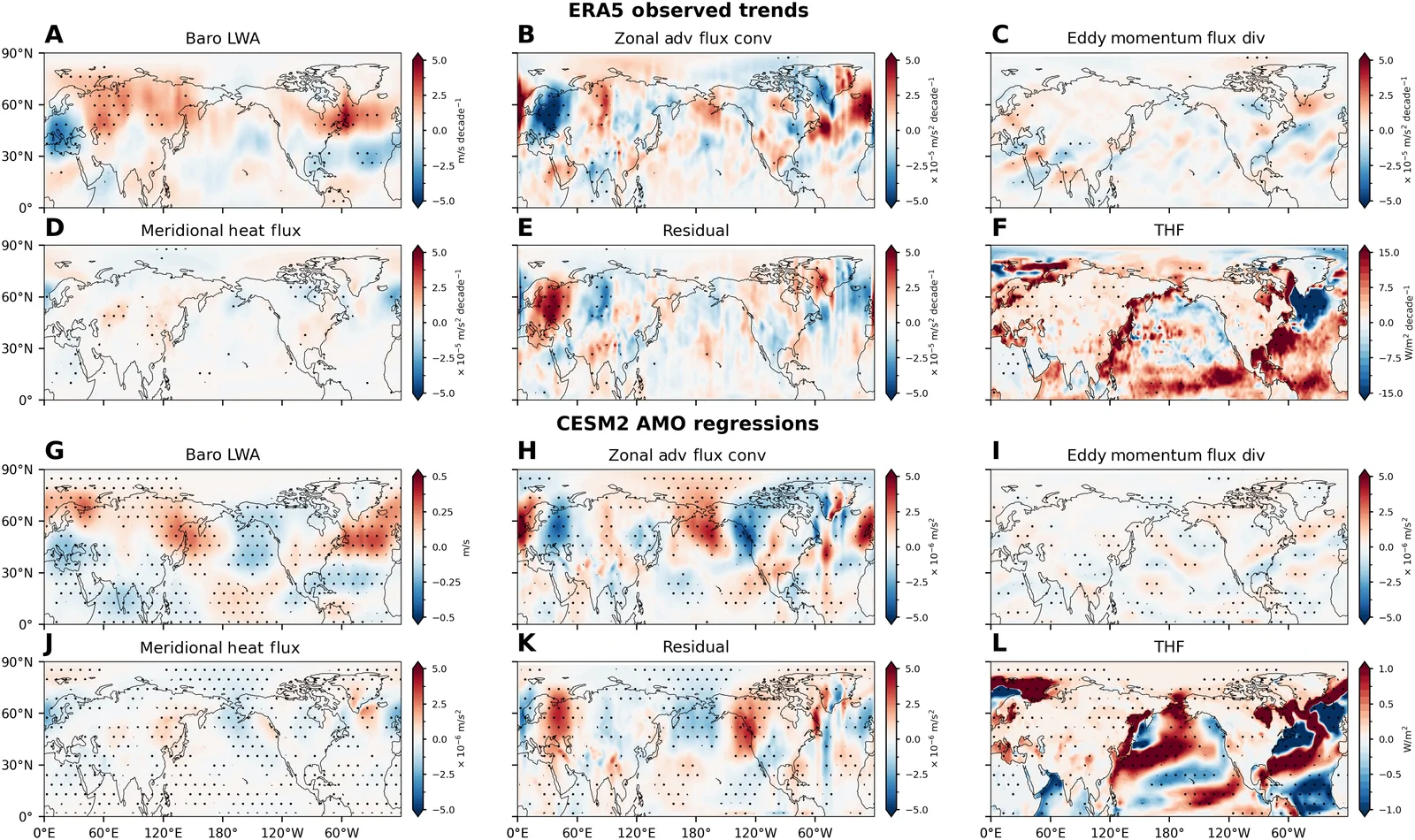
Mechanisms of the AMO in driving the LWA trends. (**A**) Trends in the quasi-geostrophic potential vorticity-based LWA in ERA5. (**B**) Trends in the zonal advective flux convergence. (**C**) Trends in eddy momentum flux divergence. (**D**) Trends in the low-level meridional heat flux. (**E**) Trends in the residual term. (**F**) Trends in turbulent heat flux. (**G** to **L**) Same as (A) to (F), but for the regression patterns associated with positive AMO in CESM2 Large Ensemble. Stippled areas indicate that the trends or regressions are significant at the 0.05 level based on the Student’s *t* test.

The QGPV-based LWA framework decomposes the LWA tendency into four terms: (i) zonal advective LWA flux convergence, (ii) meridional eddy momentum flux divergence, (iii) low-level eddy meridional heat flux, and (iv) a residual term that includes nonconservative sources and sinks such as diabatic heating, diffusion, radiative and frictional damping, and numerical errors (Materials and Methods). Over the northern North Atlantic, the enhanced LWA trend is primarily driven by anomalous zonal advective LWA flux convergence (Fig. 4B). Climatologically, this term (i) is negative in the region, whereas the low-level eddy meridional heat flux source term (iii) is positive, indicating a divergence of LWA from its source toward downstream (fig. S15, A, B, E, and F). During the study period, weakening of the westerly flow (fig. S15C) reduced its “carrying capacity” for LWA, thereby weakening the climatological divergence and producing anomalous convergence locally and anomalous divergence immediately downstream (*39*). This is also consistent with weaker westerlies that lead to an increased timescale for jet meandering (*40*). The positive LWA trend over Eurasia arises from a combination of processes (Fig. 4, B to E). In central Eurasia, zonal advective flux convergence provides the dominant contribution. In southern Eurasia, low-level meridional heat flux dominates, likely associated with enhanced cold-air outbreaks during the period, consistent with the observed strengthening of northerly winds (fig. S15D). In western and eastern Eurasia, the residual term is the largest contributor. Within this term, diabatic heating, including latent heating, radiative fluxes, and surface turbulent heat flux (THF), plays a central role. In particular, upward THF increases significantly over both regions, with the largest anomalies over adjacent coastal oceans (Fig. 4F). Such THF anomalies can modify near-surface air temperature and thereby reshape low-level temperature gradients, especially those tied to land-sea contrast and SST frontal regions, ultimately altering the background baroclinicity (*41*–*43*). Moreover, when THF-induced warming is concentrated near the surface, it can reduce static stability, thereby enhancing the Eady growth rate (*44*). The enhanced baroclinicity, as reflected by a higher Eady growth rate, promotes stronger baroclinic eddy growth, which amplifies LWA via increased eddy heat fluxes and diabatic wave sources (*45*–*48*). While these mechanisms provide a possible physical pathway linking surface thermal forcing to LWA variability, a more direct causal attribution between THF and LWA amplification requires further analysis, particularly using vertically resolved diabatic heating rates that are not available in the present dataset. Last, relative to the other terms, the contribution of meridional eddy momentum flux divergence is small, with the only notable exception being a weak positive signal over the northern North Atlantic (Fig. 4C).

To assess whether AMO-related LWA variability is dynamically consistent with the observed LWA trend pattern, we next examine the regression patterns of the QGPV LWA budget terms onto the positive AMO phase. The AMO-related regression patterns in CESM2 Large Ensemble (Fig. 4, G to L) reproduce several key features of the observed QGPV LWA budget (Fig. 4, A to F), including contributions from zonal advective LWA flux convergence over the northern North Atlantic and central Eurasia, low-level eddy meridional heat flux over southern Eurasia, and residual processes over western and eastern Eurasia. The correspondence is not exact for every budget term or region, as expected given that the observed trend reflects the combined influences of AMO-related variability, IPO-related variability, atmospheric internal variability, and possible forced components, whereas the CESM2 Large Ensemble regression isolates the model-mean linear relationship associated with AMO variability. The associated changes in THF (Fig. 4L), zonal wind (fig. S15G), and meridional wind (fig. S15H) are also broadly consistent with the observed trends. Further support comes from the CESM1 and IPSL-CM6A-LR Atlantic Pacemaker experiments, in which time-evolving North Atlantic SST anomalies are restored toward observations while the rest of the coupled climate system evolves freely (Materials and Methods). During 1989 to 2011, the CESM1 experiment produces circulation trends that are broadly consistent with both the observed circulation trends and the AMO-associated CESM2 Large Ensemble regression patterns, including weakened westerlies over the northern North Atlantic, northern North Pacific, and northern Eurasia, together with strengthened westerlies farther equatorward over the North Atlantic and North Pacific (fig. S16A; cf. fig. S15, C and G). The associated meridional wind response also captures several key features of the observed and AMO-related patterns, including anomalies over the Bering Strait, northeastern Pacific, North Atlantic, and Northern Eurasia (fig. S16B; cf. fig. S15, D and H). The IPSL-CM6A-LR experiment produces a generally weaker circulation response but similarly captures weakened 500-mb westerlies over the northern North Atlantic and northern Eurasia, together with meridional wind anomalies over the Arctic and northern Eurasia that are broadly consistent with the observed and AMO-related patterns (fig. S16, C and D). The relatively weak circulation response in the IPSL-CM6A-LR Atlantic Pacemaker experiment is also consistent with the weak and statistically insignificant LWA trend in its AMIP ensemble (fig. S4A). Previous studies have reported relatively weak atmospheric responses to tropical SST variability in IPSL-CM6A-LR, including weak El Niño–Southern Oscillation (ENSO)–related precipitation and East Asian winter monsoon responses despite relatively large ENSO SST variability (*49*, *50*). Whether IPSL-CM6A-LR similarly exhibits a systematically weak response to extratropical SST forcing, and the mechanisms underlying such a response, remain unclear and warrant further investigation. Thus, the QGPV LWA budget analysis, together with the two Atlantic Pacemaker experiments, provides process-level support for the AMO-related mechanism inferred from the AMIP, coupled large-ensemble, MCA, and AMO/IPO regression analyses. Together with these lines of evidence, these dynamical and targeted-modeling results further support the interpretation that the positive AMO phase shift made a substantial contribution to the recent increase in LWA, and hence in circulation waviness.

### Near-future circulation waviness shaped by internal variability

Previous studies have suggested that circulation waviness will likely undergo a modest decline by the end of the 21st century (*19*, *27*). Looking ahead, the near-future evolution of circulation waviness will continue to be shaped by both external forcing and internal variability, but their relative importance remains uncertain. Figure 5 shows the ensemble-mean LWA trends for 2026 to 2055 from three single-model large ensembles with 50 members each. The models disagree in both the spatial pattern and magnitude of the projected trends. For example, CESM2 Large Ensemble and MPI-ESM1-2-LR simulate an enhanced LWA over northwestern North America, whereas in EC-Earth3, the enhancement shifts eastward toward the Bering Strait. Similarly, EC-Earth3 and MPI-ESM1-2-LR simulate a weakly enhanced LWA south of Greenland, which is absent in CESM2 Large Ensemble. When averaged over the Northern Hemisphere midlatitudes, only CESM2 Large Ensemble produces a significant negative trend, while the other two models show very weak, statistically insignificant changes. Across all three ensembles, however, the intermember spread is large, compared to the ensemble-mean signal (Fig. 5D). This indicates that although intermodel differences contribute to the spread, internal variability dominates the uncertainty of near-future LWA projections.

**Fig. 5.**
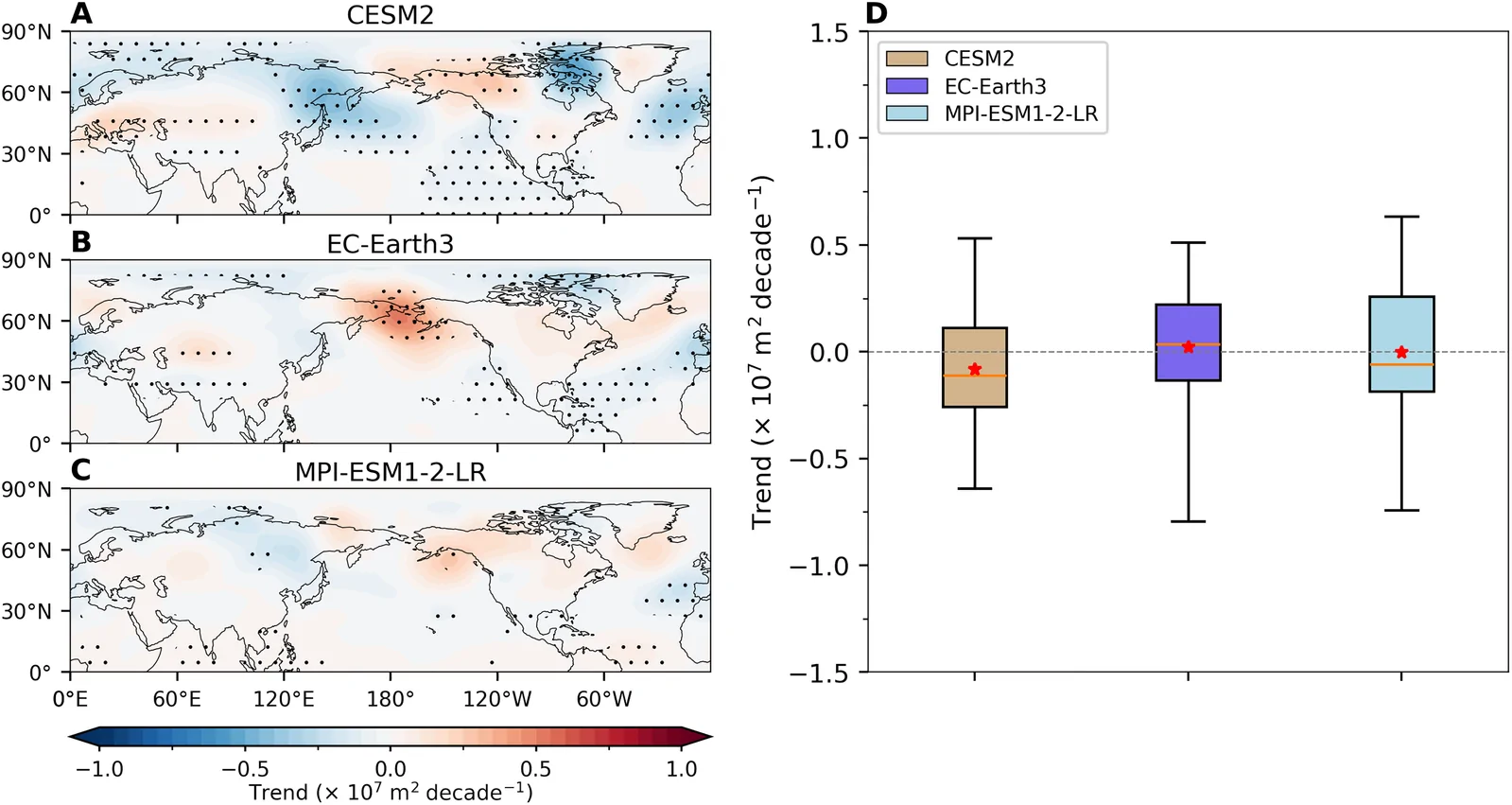
Uncertainties in the predicted LWA trends during 2026 to 2055. (**A**) Predicted ensemble mean LWA trends in CESM2 Large Ensemble. (**B**) and (**C**) are the same as (A) but for the ensemble mean trends in EC-Earth3 and MPI-ESM1-2-LR, respectively. (**D**) Average LWA trends over the Northern Hemisphere midlatitudes (40°N to 80°N) in the three model ensembles with 50 members (bars and whiskers). The orange lines (red stars) in (D) represent the ensemble median (mean). The boxes represent the 25th to 75th percentile range of the spread. The whiskers denote the maximum and minimum of the spread. The stippled areas in (A), (B), and (C) indicate trends that are significant at the 0.05 level based on the Student’s *t* test.

To further assess whether AMO and IPO variability contributes to this uncertainty, we adopt a subsampling approach similar to Cai *et al.* (*51*). For each ensemble, we identify the three members with the largest near-future AMO or IPO index trends (AMO + 3 or IPO + 3) and the three members with the smallest AMO or IPO index trends (AMO − 3 or IPO − 3), and then compare their LWA trends. Despite the influence of atmospheric internal variability and other sources of oceanic variability, the AMO + 3 members consistently show enhanced LWA trends over northeastern Eurasia relative to the AMO − 3 members across all three ensembles (fig. S17, A, C, and E), consistent with the observed trend pattern. In CESM2 Large Ensemble and EC-Earth3, the AMO + 3 members also exhibit stronger LWA trends over the northern North Atlantic. When averaged over the Northern Hemisphere midlatitudes, the AMO + 3 members in CESM2 Large Ensemble and MPI-ESM1-2-LR produce significantly positive LWA trends that are at least an order of magnitude larger than their respective ensemble-mean trends shown in Fig. 5. For the IPO, the IPO + 3 members consistently show stronger positive LWA trends over the northern North Pacific relative to the IPO − 3 members (fig. S17, B, D, and F), consistent with the IPO-associated LWA patterns identified from the historical large-ensemble analyses (Fig. 3B and fig. S12, B, D, F, H, and J). Averaged over the Northern Hemisphere midlatitudes, the IPO + 3 members in EC-Earth3 and MPI-ESM1-2-LR also produce significantly positive LWA trends that are at least an order of magnitude larger than their respective ensemble-mean trends. These subsampling results suggest that the future trajectories of the AMO and IPO can strongly modulate near-term changes in midlatitude LWA. Given the demonstrated influence of the IPO and AMO on multidecadal LWA variability, the mechanisms identified in this study offer a pathway for reducing uncertainty and improving projections of future circulation waviness.

## DISCUSSION

The short-term increase in circulation waviness from 1989 to 2011 has sparked vigorous debate over whether it reflects AA, mostly due to external forcing, or internal variability. Using model simulations, statistical analyses, and dynamical theory, we show that the positive phase shift of the AMO contributed prominently to the observed waviness trend during the study period. Because recent AMO variability may itself include both internally generated and externally forced components (*52*, *53*), this result does not rule out a forced contribution. Instead, it points to the important role of AMO-related oceanic variability and suggests that internal variability may have played a potentially critical role in shaping the observed short-term waviness trend. This interpretation is broadly consistent with Sun *et al.* (*38*), who examined boreal summer circulation waviness and found that enhanced summertime jet waviness over the Northern Hemisphere during recent decades was closely linked to tropical eastern Pacific cooling, suppressed tropical convection, and associated Rossby-wave propagation, while the role of AA appeared limited. Although their study focused on summer and emphasized tropical Pacific forcing, whereas our study focuses on winter and highlights the role of North Atlantic SST variability associated with the AMO, both studies suggest that boundary SST variability can play an important role in shaping midlatitude circulation waviness and that AA alone is unlikely to fully explain the observed waviness changes. However, compared to observations, model experiments, even when forced with observed SST and sea-ice variability, often fail to reproduce waviness trends of comparable magnitude (Fig. 1 and figs. S2 and S4). This discrepancy is due to two possible causes: (i) atmospheric internal variability also contributed positively to the observed trend, but the models underestimate the amplitude and/or spatial structure of the relevant internal variability, and/or (ii) models underestimate the sensitivity of circulation waviness to the AMO, consistent with the weak signal-to-noise ratio in the response to multidecadal variability documented in previous studies (*32*–*34*). Models display varying sensitivity to the AMO phase shift (fig. S12). The extreme magnitude of the observed trend therefore remains an open question for future research.

One caveat is that North Atlantic decadal variability may be partly influenced by atmospheric circulation (*54*). In the coupled climate system, atmospheric circulation anomalies can affect North Atlantic SSTs through surface heat fluxes, wind-driven ocean processes, and ocean mixing. Thus, part of the observed Atlantic SST signal prescribed in AMIP-type experiments may contain the imprint of prior atmospheric circulation variability. If such circulation-imprinted SST anomalies subsequently feed back onto the atmosphere in a way that projects onto the original circulation pattern, AMIP simulations may reproduce part of the observed waviness response without necessarily implying that the AMO was the sole initial driver. We therefore interpret the AMIP results as evidence that observed ocean boundary conditions are capable of producing an LWA response that shares key regional features with the observed trend, rather than as definitive proof of one-way causality from AMO-related SST anomalies to LWA. The role of AMO-related variability is instead inferred from the combined evidence provided by the AMIP simulations, coupled large-ensemble analyses, MCA, AMO/IPO regressions, and LWA-budget diagnostics.

Nevertheless, this study provides evidence for a prominent role of the AMO in driving the short-term increase in circulation waviness during 1989 to 2011, thereby further clarifying the role of AA in midlatitude circulation variability. Although our results suggest that AA is unlikely to be the primary driver of the decadal waviness increase examined here, this does not imply that AA and Arctic sea-ice loss exert only weak influences on midlatitude weather and climate. A substantial body of work shows that AA and sea-ice loss can modulate other aspects of midlatitude dynamics, such as the meridional position and zonal extent of the eddy-driven jet, thereby affecting midlatitude variability and weather extremes (*15*, *24*, *55*). In addition, thermodynamic pathways associated with Arctic change can amplify midlatitude summertime heat extremes (*56*). Together, these findings indicate that AA has been, and will likely remain, an important contributor to midlatitude climate and extremes, even if its role in the wintertime waviness changes examined here is secondary.

Our results also point to the AMO as a potential source of predictability for multidecadal changes in circulation waviness. Given its 60- to 80-year cycle and its predominantly positive phase during the past four decades (fig. S3C), if the AMO starts to transition toward a negative phase in the coming decades, it could favor less wavy circulations over the Northern Hemisphere, particularly Eurasia, with profound implications for less wintertime extremes.

## MATERIALS AND METHODS

### Observational datasets

Observational analyses are based on five reanalysis products. The primary results in the main text use the ERA5 (*57*). To test the robustness of the LWA trends, we also analyze four additional reanalyses: the Modern-Era Retrospective analysis for Research and Applications, version 2 (MERRA-2) (*58*), the Japanese 55-year Reanalysis (JRA-55) (*59*), the ECMWF Interim Reanalysis (ERA-Interim) (*60*), and the Climate Forecast System Reanalysis (CFSR) (*61*), as shown in fig. S1. All reanalyses were interpolated to a common 2° × 2° grid before analysis. Daily data were derived from 6-hourly fields at 00, 06, 12, and 18 UTC. Following previous studies (*2*, *4*), we analyze the cold season (December to February) during 1989 to 2011, selected because it exhibits the strongest multidecadal LWA trend over Northern Hemisphere midlatitudes (40° to 80°N) relative to any other period exceeding 20 years in length (fig. S18). SST data are taken from the Met Office Hadley Centre dataset (HadISST) (*62*).

### Model simulations

Six initial-condition large ensembles are used to quantify the influence of anthropogenic forcing on LWA trends and to test the robustness of the results: CESM2 (50 members), EC-Earth3 (50 members), MPI-ESM1-2-LR (50 members), IPSL-CM6A-LR (33 members), NorCPM1 (30 members), and MIROC6 (10 members). The available historical coverage differs among ensembles: 1850 to 2014 for CESM2 Large Ensemble and MPI-ESM1-2-LR, 1970 to 2014 for EC-Earth3, and 1950 to 2014 for IPSL-CM6A-LR and NorCPM1. The primary results shown in Figs. 1 and 4 are based on the CESM2 Large Ensemble (*63*). CESM2 Large Ensemble consists of 100 members in total, organized into two 50-member subensembles that differ only in their biomass burning (BMB) aerosol forcing (CMIP6 BMB versus smoothed CMIP6 BMB). Here, we use the subensemble with smoothed BMB forcing, as previous studies have shown that it better reproduces Arctic climate variability (*64*, *65*). All coupled ensembles are integrated with CMIP6 historical forcing over their respective historical periods and SSP370 forcing thereafter. To investigate the role of oceanic internal variability, we also analyze five AMIP-type ensembles driven by observed SST and sea ice, each with at least 10 members: CESM2 (10 members), IPSL-CM6A-LR (22 members), MIROC6 (10 members), NorCPM1 (10 members), and IPSL-CM6A-MR1 (10 members). The CESM2 AMIP ensemble provides the primary results in the main text. As with the coupled ensembles, the AMIP simulations are driven by historical forcing over the study period. To further assess the circulation response to North Atlantic SST variability, we also analyze two 10-member Atlantic Pacemaker ensembles from CESM1 and IPSL-CM6A-LR (*66*–*68*). In these experiments, time-evolving SST anomalies over the North Atlantic are nudged toward observations, while the rest of the coupled climate system is allowed to evolve freely. We use these pacemaker experiments as additional targeted-modeling tests of whether observed North Atlantic SST variability can influence large-scale atmospheric circulation during 1989 to 2011. For this analysis, we use monthly output at each model’s native resolution. For the coupled and AMIP ensembles, daily outputs are used and regridded to a common 2° × 2° resolution before analysis.

### LWA framework

LWA is used to quantify the waviness of planetary-scale circulation. Following previous studies (*29*, *30*), LWA measures the meridional displacement of geopotential height or QGPV contours relative to a zonally symmetric reference state, thereby providing an objective metric of wave amplitude. LWA has been shown to capture large-scale waviness associated with troughs and ridges, low-pressure systems, and atmospheric blocking (*30*, *48*, *69*). We mostly use LWA based on daily 500-hPa geopotential height because of its simplicity and computational efficiency, and to enable a direct comparison with Blackport and Screen (*4*). At each longitude (λ), latitude (ɸ), height (*z*), and time (*t*), LWA is defined as

A(λ,ϕ,z,t)=acosϕ∫0Δϕqe(λ,ϕ,ϕ′,z,t)cos(ϕ+ϕ′)dϕ′ (1)

where a is the radius of Earth, Δϕ is the meridional displacement of the reference contour ($q_{\text{ref}}$), and $q_{e}$ is the eddy component of geopotential height or QGPV given by

$$q_{e}\left(\lambda ,\phi ,\phi^{\prime },z,t\right)=q\left(\lambda ,\phi +\phi^{\prime },z,t\right)-q_{\text{ref}}\left(\phi ,z,t\right)$$ (2)

Here, $q_{\text{ref}}$ is defined by adjusting the height contours to a zonally symmetric state while conserving the poleward enclosed area.

Although computationally simple, geopotential height–based LWA lacks a dynamical underpinning, preventing mechanistic analysis of the processes driving its changes. To address this, Huang and Nakamura (*29*) developed a prognostic equation for LWA defined in terms of QGPV, which links the LWA tendency to individual dynamical processes

$$\begin{array}{c} \frac{\partial }{\partial t}\langle A\rangle \text{cosϕ}\\ =-\frac{1}{a\text{cosϕ}}\frac{\partial \langle F_{\lambda }\rangle }{\partial \lambda }+\frac{1}{a\text{cosϕ}}\frac{\partial }{\partial \phi }\langle u_{e}v_{e}{\text{cos}}^{2}\text{ϕ}\rangle +\frac{f\text{cosϕ}}{H}{\left(\frac{v_{e}\theta_{e}}{\partial \tilde{\theta }/\partial z}\right)}_{z=0}+\langle \dot{A}\rangle \text{cosϕ} \end{array}$$ (3)

where 〈 〉 denotes density-weighted vertical average, $F_{\lambda }$ is the zonal LWA flux, f is the Coriolis parameter, and $u_{e}$, $v_{e}$, and $\theta_{e}$ are the eddy components of zonal wind, meridional wind, and potential temperature, respectively. $\tilde{\theta }$ is the area-weighted hemispheric average of potential temperature. The first two terms represent zonal and meridional LWA flux convergence, the third term corresponds to low-level eddy meridional heat flux, and the fourth term, which is the residual, includes nonconservative sources and sinks such as diabatic heating, diffusion, radiative and frictional damping, and numerical errors.

In this study, the calculation of the QGPV-based LWA budget terms (Eq. 3) is performed using daily zonal wind, meridional wind, and temperature at eight pressure levels (1000, 850, 700, 500, 200, 100, 50, and 10 hPa). These levels were chosen because they are the only vertical levels archived in CESM2 Large Ensemble. The algorithm for calculating each budget term is described in Huang *et al.* (*70*). The computation of QGPV-based LWA is nearly identical to that of 500-hPa geopotential height–based LWA (Eq. 2), except that a negative sign is applied due to the opposite meridional gradient: QGPV increases with latitude in the Northern Hemisphere, whereas geopotential height decreases. For a more detailed description of the LWA framework, readers are referred to Huang and Nakamura (*29*).

### IPO and AMO definition

Following Tokinaga *et al.* (*37*) and Ma *et al.* (*71*), the IPO is characterized using the leading EOF of detrended SST anomalies across the Pacific basin (120°E to 70°W, 50°S to 60°N). The IPO index is taken as the principal component associated with this mode, smoothed with a 7-year running mean. The AMO is defined as the 7-year running mean of detrended SST anomalies averaged over the North Atlantic (60°W to 0°, equator to 70°N). In the observational record, detrending is performed by subtracting the linear trend over the full HadISST period (1870 to 2025). In model simulations, detrending is achieved by removing the ensemble-mean time series (representing the externally forced signal) from each individual member.

### Adjustment of LWA based on observational AMO

To further demonstrate the role of the AMO in modulating LWA variability, we apply a statistical adjustment to the simulations following the method of Salzmann and Cherian (*72*). Specifically, we replace the component of simulated LWA variability that is linearly associated with the simulated AMO with the corresponding variability associated with the observed AMO. This produces adjusted LWA time series averaged over Northern Hemisphere midlatitudes (40° to 80°N), expressed as

LWAadj(i)=LWAforced+LWAinternal(i)−LWAsimulated_AMO(i)+LWAobserved_AMO (4)

where ${\text{LWA}}_{\text{forced}}$ is the ensemble-mean time series representing the forced response, and ${\text{LWA}}_{\text{internal}}\left(i\right)$ is the residual variability in member i after removing the forced component. The term ${\text{LWA}}_{\text{simulated}\_\text{AMO}}\left(i\right)$ represents the variability linearly associated with the simulated AMO, while ${\text{LWA}}_{\text{observed}\_\text{AMO}}\left(i\right)$ represents the variability linearly associated with the observed AMO.

More explicitly, these AMO-related components are estimated as

$${\text{LWA}}_{\text{simulated}\_\text{AMO}}\left(i\right)=r_{\text{LWA},\text{AMO}}\left(i\right)\text{AMO}\left(i\right)$$ (5)

$${\text{LWA}}_{\text{observed}\_\text{AMO}}\left(i\right)=r_{\text{LWA},\text{AMO}}\left(i\right){\text{AMO}}_{\text{observed}}$$ (6)

where $r_{\text{LWA},\text{AMO}}\left(i\right)$ is the regression coefficient of LWA onto the AMO index in member i. Here, AMO(i) is the simulated AMO index in member i, while ${\text{AMO}}_{\text{observed}}$ is the observed AMO index.
