# Supplementary material for: Increases in Northern Hemisphere winter circulation waviness over 1989 to 2011 driven by decadal oceanic variability
Source: Sci Adv. 2026 Nov 13;12(46):eaeg2637. doi: 10.1126/sciadv.aeg2637 (PMC13763854; doi:10.1126/sciadv.aeg2637)
Supplement: Supplementary file 1 [file sciadv.aeg2637_sm.pdf]

Science Advances
AAAS

Supplementary Materials for

Increases in Northern Hemisphere winter circulation waviness over 1989 to 2011 driven by decadal oceanic variability

Weiming Ma *et al.*

Corresponding author: Weiming Ma, weiming.ma@pnnl.gov; Hailong Wang, hailong.wang@pnnl.gov

Sci. Adv. **12**, eaeg2637 (2026)
DOI: 10.1126/sciadv.aeg2637

This PDF file includes:

Figs. S1 to S18

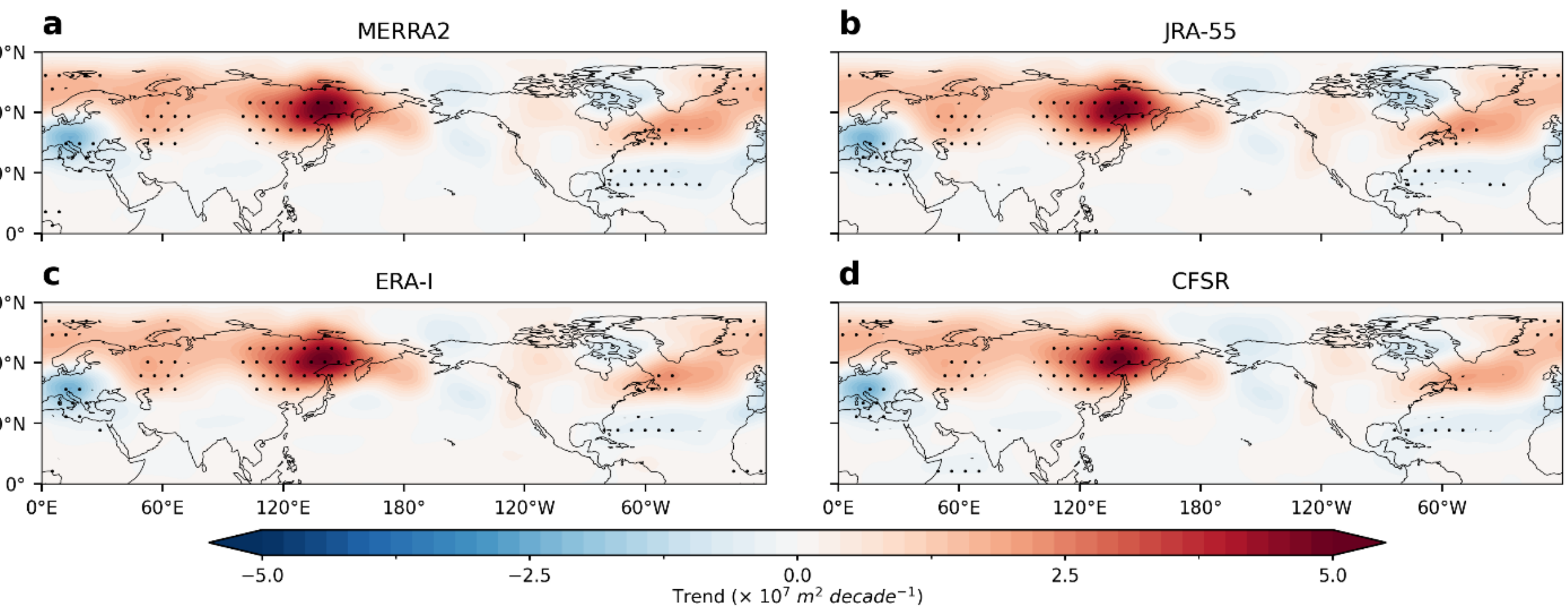

Fig. S1. Observed LWA trends in different reanalyses. Observed LWA trends in **a**, MERRA2. **b**, JRA-55. **c**, ERA-I. **d**, CFSR. The stippled areas indicate trends that are significant at the 0.05 level based on the Student's t-test.

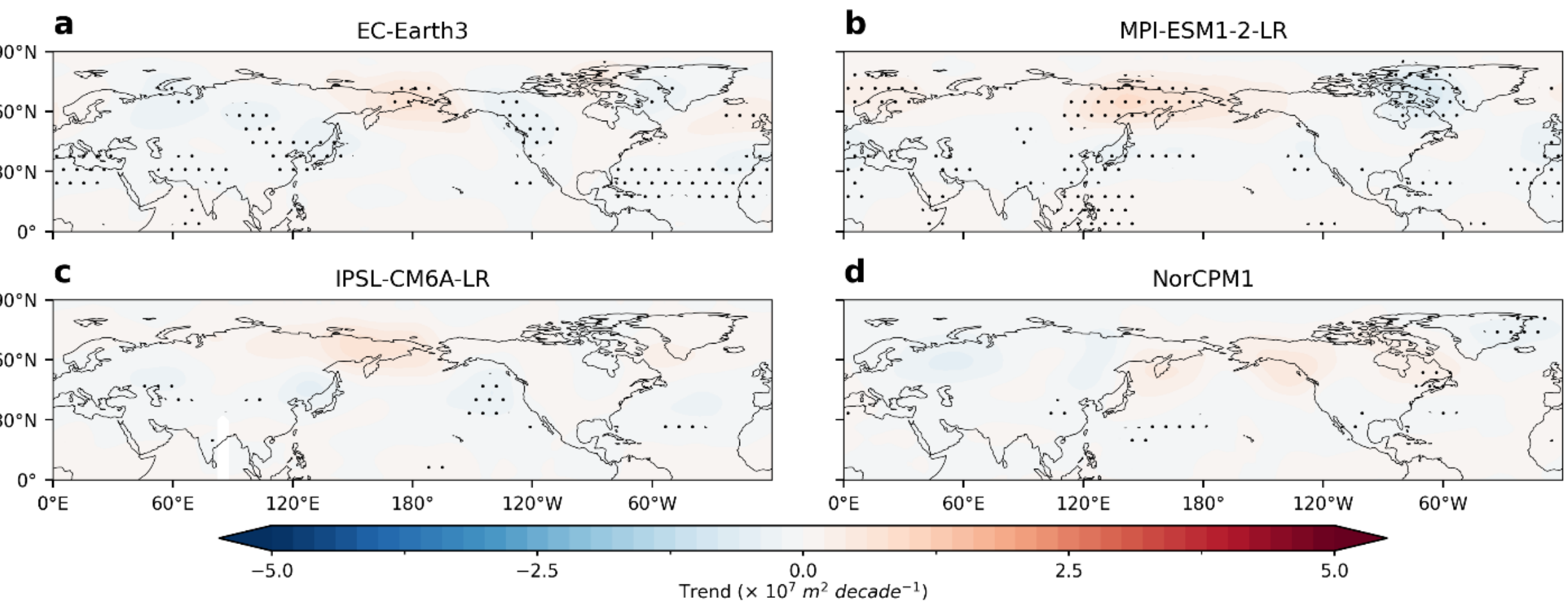

Fig. S2. Ensemble mean trends in LWA from 1989-2011 based on four CMIP6 models. a, Ensemble mean LWA trends in EC-Earth3 (50 members). **b**, **c**, and **d** are same as **a**, but for MPI-ESM1-2-LR (50 members), IPSL-CM6A-LR (33 members), and NorCPM1 (30 members), respectively. The stippled areas indicate trends that are significant at the 0.05 level based on the Student's t-test.

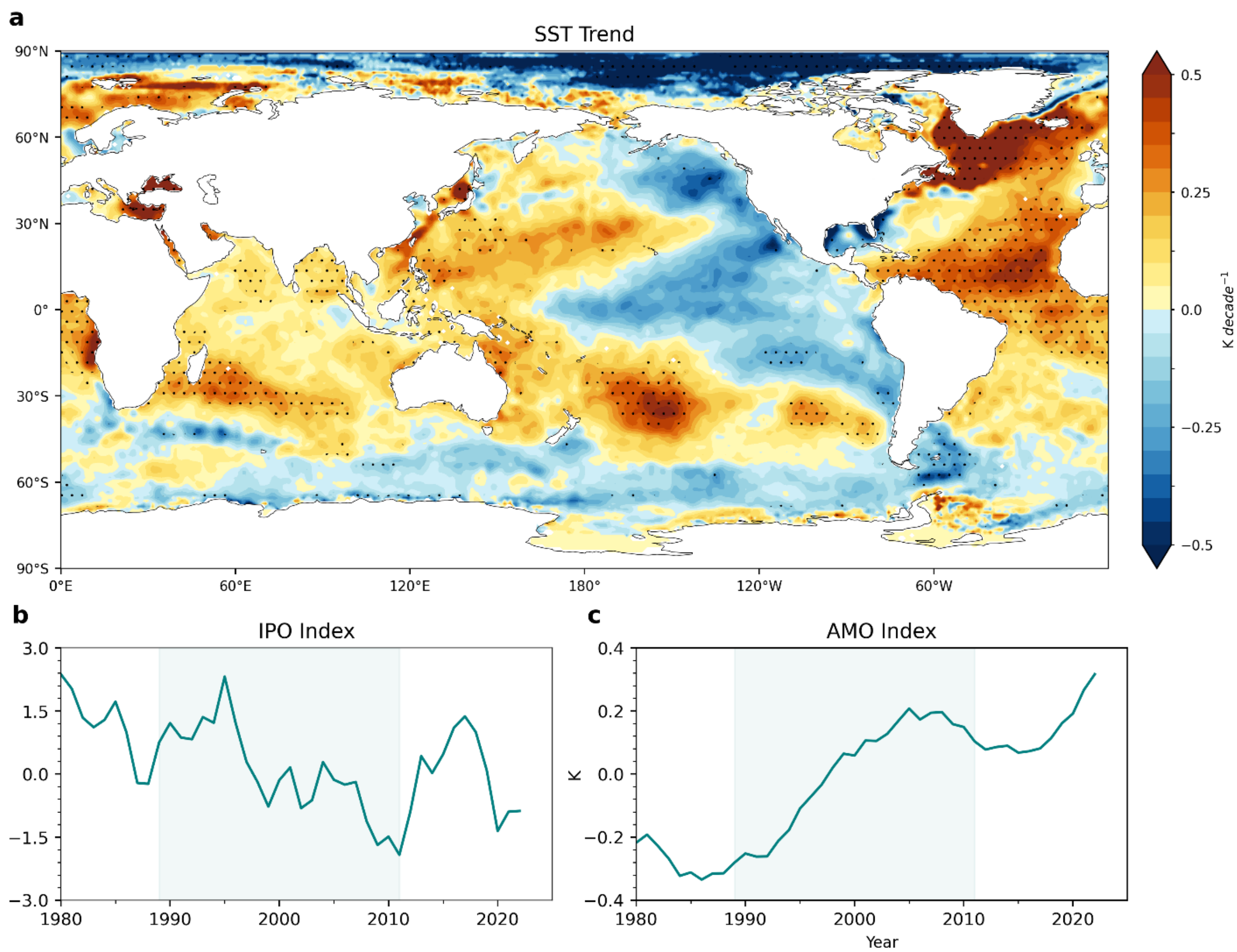

Fig. S3. Observed sea surface temperature (SST) trend, Interdecadal Pacific Oscillation (IPO), and Atlantic Multidecadal Oscillation (AMO) time series. a, Observed SST trend from 1989 to 2011. **b**, Observed IPO index from 1980 to 2022. The shading highlights the study period from 1989 to 2011. **c**, Same as **b**, but for the AMO index. The stippled areas in **a** indicate trends that are significant at the 0.05 level based on the Student's t-test.

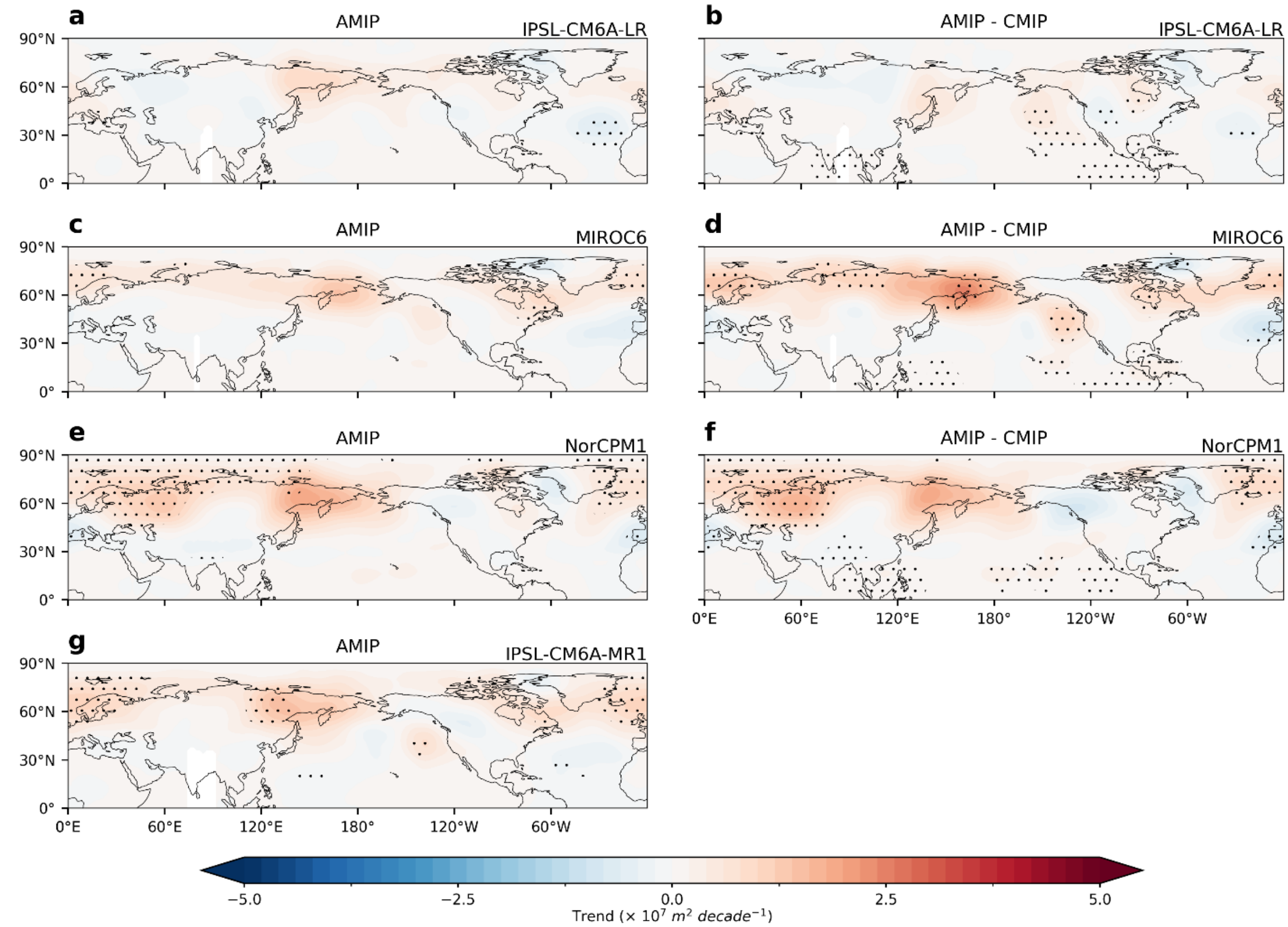

Fig. S4. Ensemble mean trends in LWA from 1989-2011 based on four AMIP models. a, Ensemble mean LWA trends in IPSL-CM6A-LR (22 members). **b**, Difference in ensemble mean LWA trends between the AMIP ensemble and the corresponding CMIP ensemble for IPSL-CM6A-LR (33 members). **c**, **e**, and **g** are same as **a**, but for MIROC6 (10 members), NorCPM1 (10 members), and IPSL-CM6A-MR1 (10 members), respectively. **d** and **f** are same as **b**, but for the ensemble mean difference in LWA trends for MIROC6 (10 members) and NorCPM1 (30 members). The corresponding CMIP ensemble for IPSL-CM6A-MR1 is not available, so the difference between AMIP ensemble and CMIP ensemble for this model is not shown. The stippled areas indicate trends or trend difference that are significant at the 0.05 level based on the Student's t-test.

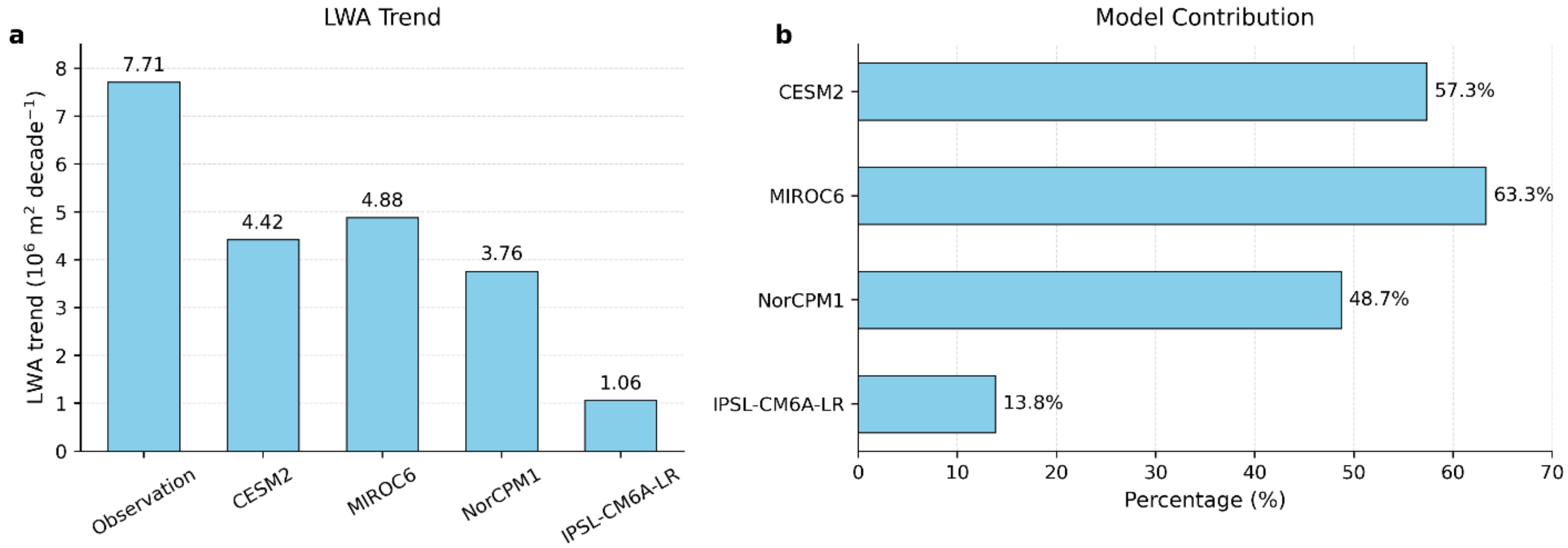

Fig. S5. Contribution of observed oceanic variability to the observed LWA trend.
a, Area-mean LWA trends over the Northern Hemisphere midlatitudes (40°N–80°N) in observations and in the AMIP–CMIP ensemble-mean difference for CESM2, MIROC6, NorCPM1, and IPSL-CM6A-LR. The AMIP–CMIP difference is used to estimate the component of the LWA trend driven by observed oceanic variability. **b,** Fractional contribution of observed oceanic variability to the observed Northern Hemisphere midlatitude LWA trend in each model, calculated as the ratio of the AMIP–CMIP LWA trend difference to the observed LWA trend.

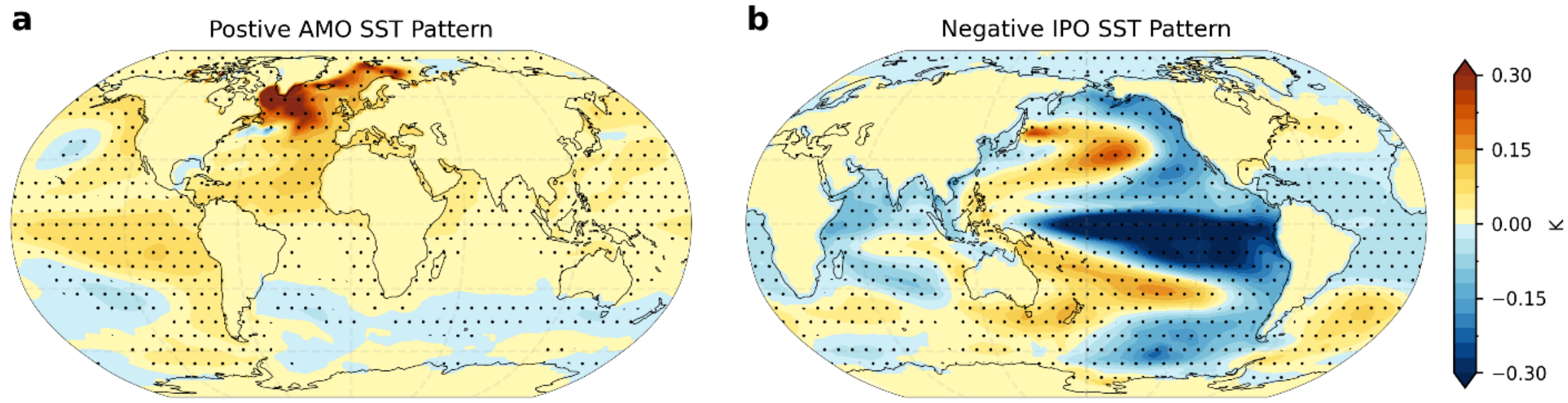

Fig. S6. Sea surface temperature (SST) pattern associated with Atlantic Multidecadal Oscillation (AMO) and Interdecadal Pacific Oscillation (IPO) in CESM2 Large Ensemble. **a**, SST regression pattern associated with positive AMO. **b**, SST regression pattern associated with negative IPO. Stippled areas indicate that regressions are significant at the 0.05 level based on the Student's *t*-test.

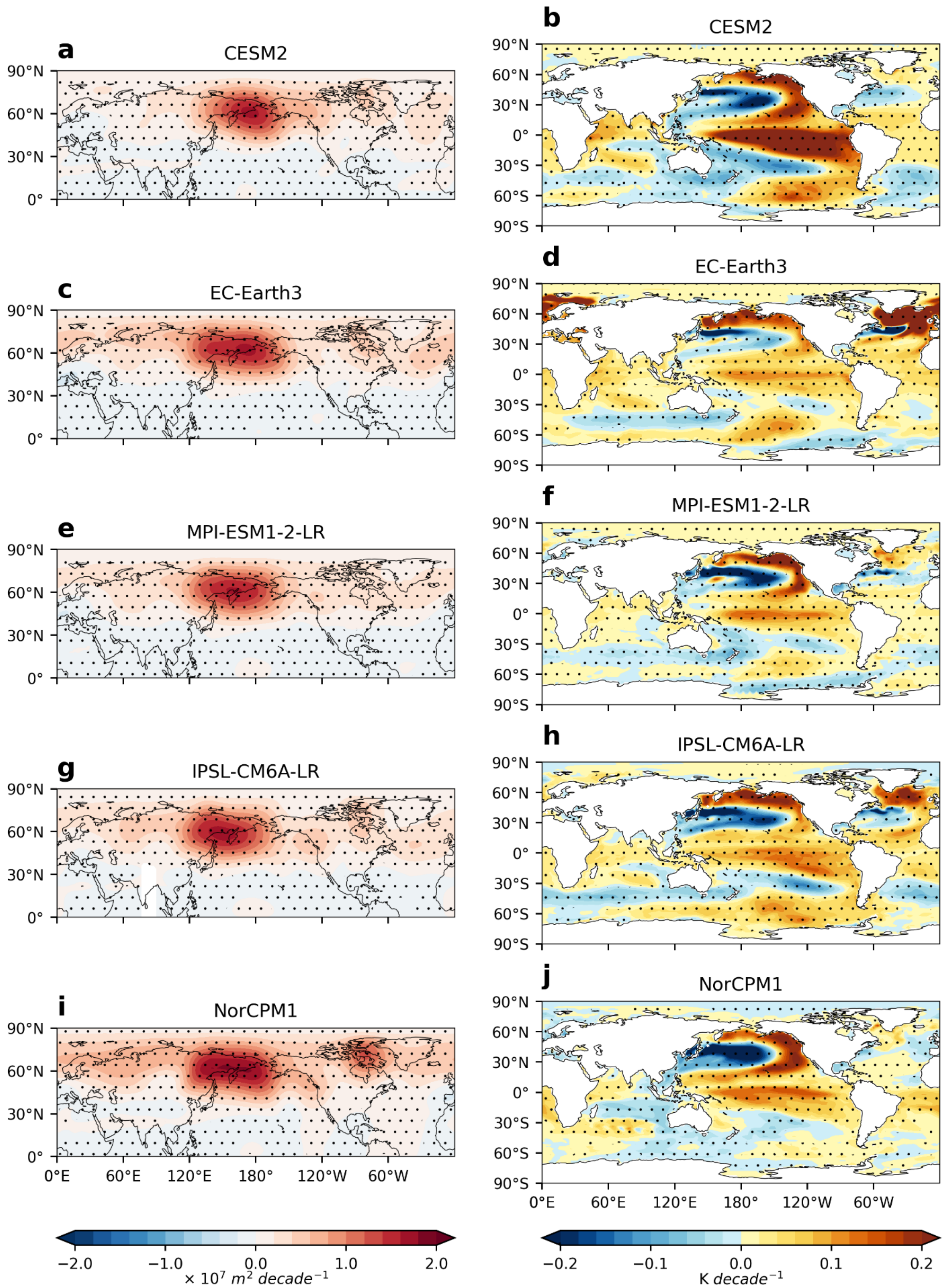

a
CESM2
b
CESM2
c
EC-Earth3
d
EC-Earth3
e
MPI-ESM1-2-LR
f
MPI-ESM1-2-LR
g
IPSL-CM6A-LR
h
IPSL-CM6A-LR
i
NorCPM1
j
NorCPM1
90°N
60°N
30°N
0°
30°S
60°S
90°S
0°E
60°E
120°E
180°
120°W
60°W
−2.0
−1.0
0.0
1.0
2.0
× 10^7 m^2 decade^−1
−0.2
−0.1
0.0
0.1
0.2
K decade^−1

Fig. S7. First mode of maximum covariance analysis (MCA) between the internally generated LWA trends and sea surface temperature (SST) trends. a, LWA trend pattern for CESM2 Large Ensemble. **b**, SST trend pattern for CESM2 Large Ensemble. **c**, **d** Same as **a** and **b**, but for EC-Earth3. **e**, **f** Same as **a** and **b**, but for MPI-ESM1-2-LR. **g**, **h** Same as **a** and **b**, but for IPSL-CM6A-LR. **i**, **j** Same as **a** and **b**, but for NorCPM1. The fraction of squared covariance explained by the first MCA mode is 65.7% for CESM2 Large Ensemble, 68.2% for EC-Earth3, 72.4% for MPI-ESM1-2-LR, 61.4% for IPSL-CM6A-LR, and 72.6% for NorCPM1. Stippled areas indicate that regressions are significant at the 0.05 level based on the Student's *t*-test.

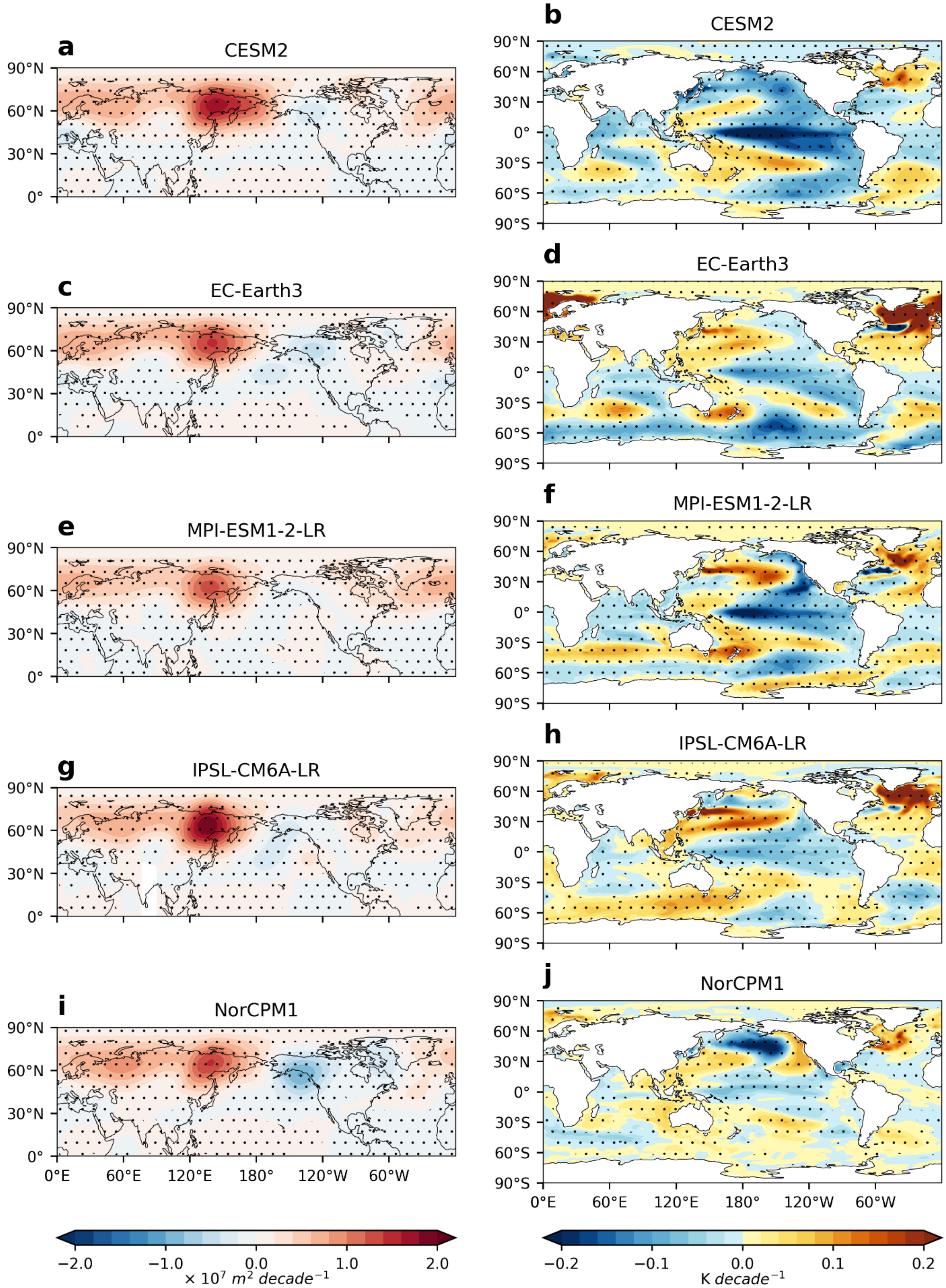
a
CESM2
b
CESM2
c
EC-Earth3
d
EC-Earth3
e
MPI-ESM1-2-LR
f
MPI-ESM1-2-LR
g
IPSL-CM6A-LR
h
IPSL-CM6A-LR
i
NorCPM1
j
NorCPM1
90°N
60°N
30°N
0°
30°S
60°S
90°S
0°E
60°E
120°E
180°
120°W
60°W
−2.0
−1.0
0.0
1.0
2.0
× 10^7 m^2 decade^−1
−0.2
−0.1
0.0
0.1
0.2
K decade^−1

Fig. S8. Second mode of maximum covariance analysis (MCA) between the internally generated LWA trends and sea surface temperature (SST) trends. a, LWA trend pattern for CESM2 Large Ensemble. **b**, SST trend pattern for CESM2 Large Ensemble. **c**, **d** Same as **a** and **b**, but for EC-Earth3. **e**, **f** Same as **a** and **b**, but for MPI-ESM1-2-LR. **g**, **h** Same as **a** and **b**, but for IPSL-CM6A-LR. **i**, **j** Same as **a** and **b**, but for NorCPM1. The fraction of squared covariance explained by the second MCA mode is 17.9% for CESM2 Large Ensemble, 10.0% for EC-Earth3, 10.8% for MPI-ESM1-2-LR, 12.8% for IPSL-CM6A-LR, and 6.3% for NorCPM1. Note that in NorCPM1, the corresponding pattern is captured by the third MCA mode rather than the second. Stippled areas indicate that regressions are significant at the 0.05 level based on the Student's *t*-test.

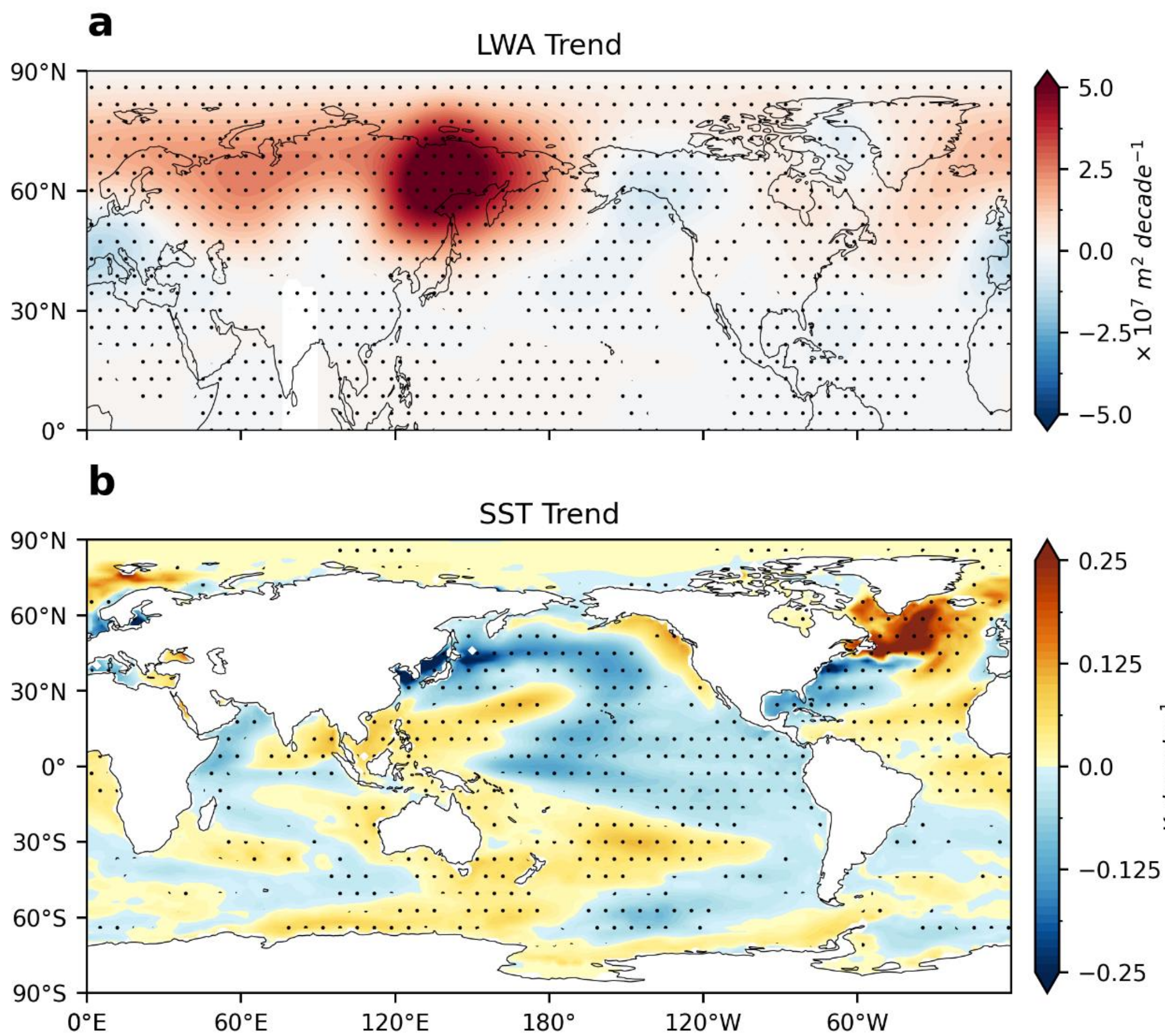

Fig. S9. Fingerprint analysis of internally generated LWA and SST trends associated with the observed LWA trend pattern. **a**, Difference in ensemble-mean LWA trends between pseudo-ensemble members whose internally generated LWA trend patterns are positively correlated with the observed LWA trend pattern and those whose patterns are negatively correlated with the observed trend pattern. **b**, Same as **a**, but for SST trends. Positive- and negative-correlation members are defined using spatial correlations ≥ 0.5 and ≤ −0.5, respectively, over the Northern Hemisphere midlatitudes (40°–80°N). Stippling denotes grid points where at least 4 of 5 ensembles agree on the sign of the multi-ensemble-mean pattern.

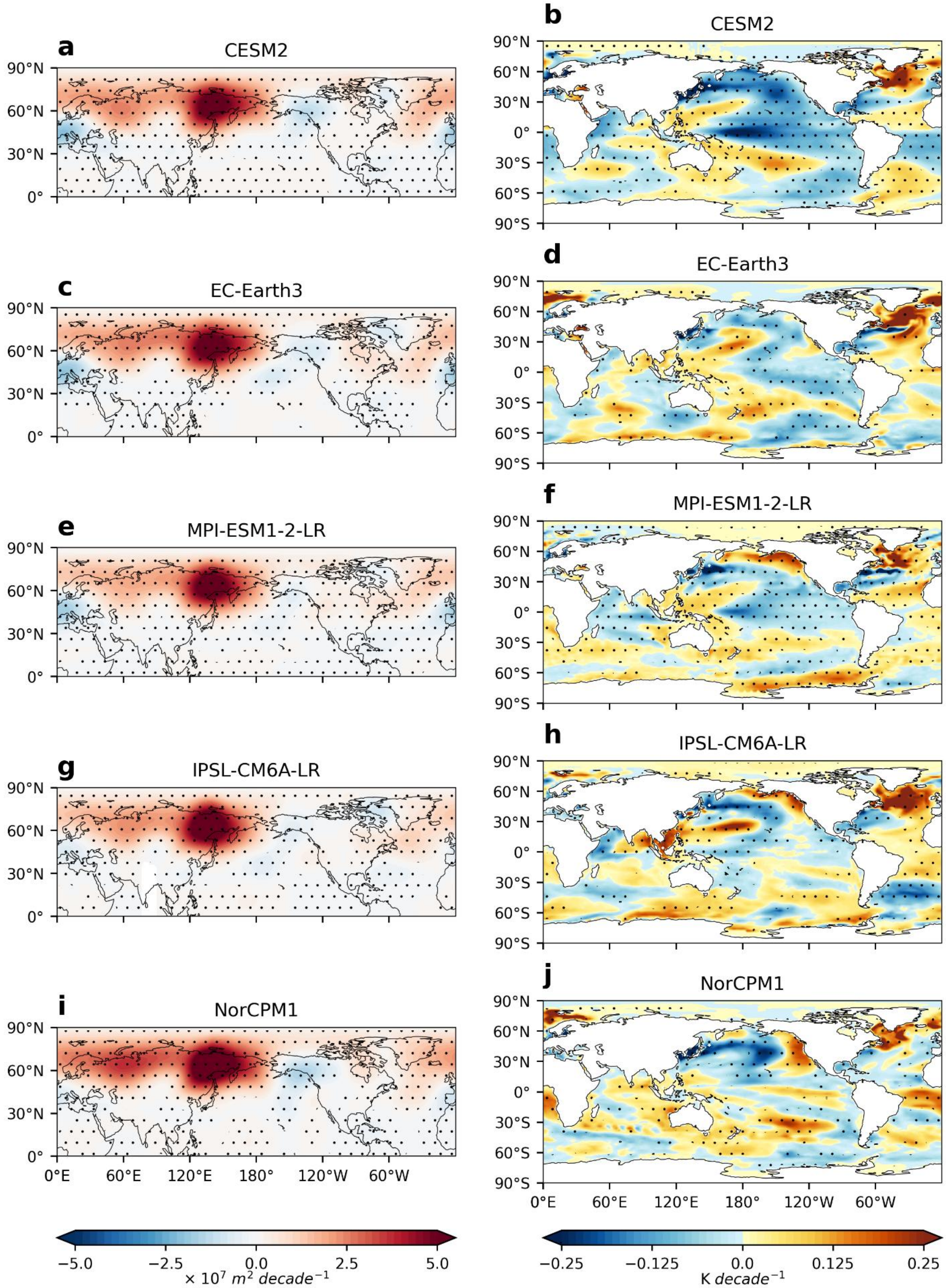

a
CESM2
b
CESM2
c
EC-Earth3
d
EC-Earth3
e
MPI-ESM1-2-LR
f
MPI-ESM1-2-LR
g
IPSL-CM6A-LR
h
IPSL-CM6A-LR
i
NorCPM1
j
NorCPM1
90°N
60°N
30°N
0°
30°S
60°S
90°S
0°E
60°E
120°E
180°
120°W
60°W
−5.0
−2.5
0.0
2.5
5.0
× 10^7 m^2 $decade^{-1}$
−0.25
−0.125
0.0
0.125
0.25
K $decade^{-1}$

Fig. S10. Same as Fig. S9, but for individual model pseudo-ensembles. **a**, **c**, **e**, **g**, and **i** show LWA trend differences between positive- and negative-correlation members in CESM2 Large Ensemble, EC-Earth3, MPI-ESM1-2-LR, IPSL-CM6A-LR, and NorCPM1, respectively. **b**, **d**, **f**, **h**, and **j** show the corresponding SST trend differences. Stippling indicates regions where the composite differences are statistically significant at the 0.05 level based on Student's t-test.

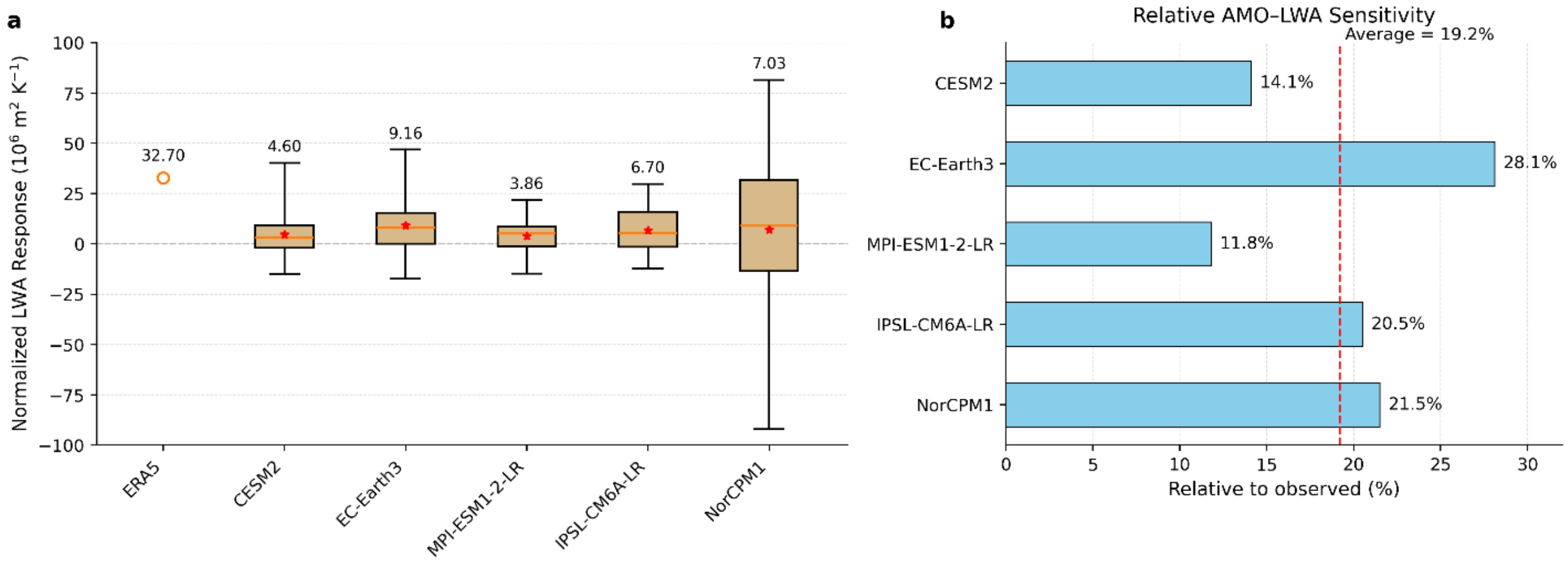

Fig. S11. Model-simulated AMO-associated LWA response relative to the observed AMO-normalized LWA trend. a, Area-mean LWA trend over the Northern Hemisphere midlatitudes (40°N–80°N) in observations, normalized by the observed AMO trend, together with the AMO–LWA sensitivities calculated separately for individual members of five model ensembles by regressing area-mean LWA variability onto the unstandardized AMO index of the same member. Results are shown for CESM2, EC-Earth3, MPI-ESM1-2-LR, IPSL-CM6A-LR, and NorCPM1. The open orange circle denotes the observed AMO-normalized LWA trend, while the boxplots show the distributions of member-specific AMO–LWA sensitivities. The orange horizontal lines and red stars denote the ensemble medians and means, respectively; boxes indicate the 25th–75th percentile range, and whiskers indicate the minimum and maximum values across ensemble members. Numbers above the open circle and boxplots indicate the observed AMO-normalized LWA trend and the ensemble-mean AMO–LWA sensitivities, respectively, in units of 10^6 m^2 K^{-1}. **b,** Ratio of the ensemble-mean AMO–LWA sensitivity in each model to the observed AMO-normalized LWA trend, expressed as a percentage. The red dashed line denotes the multi-model mean ratio.

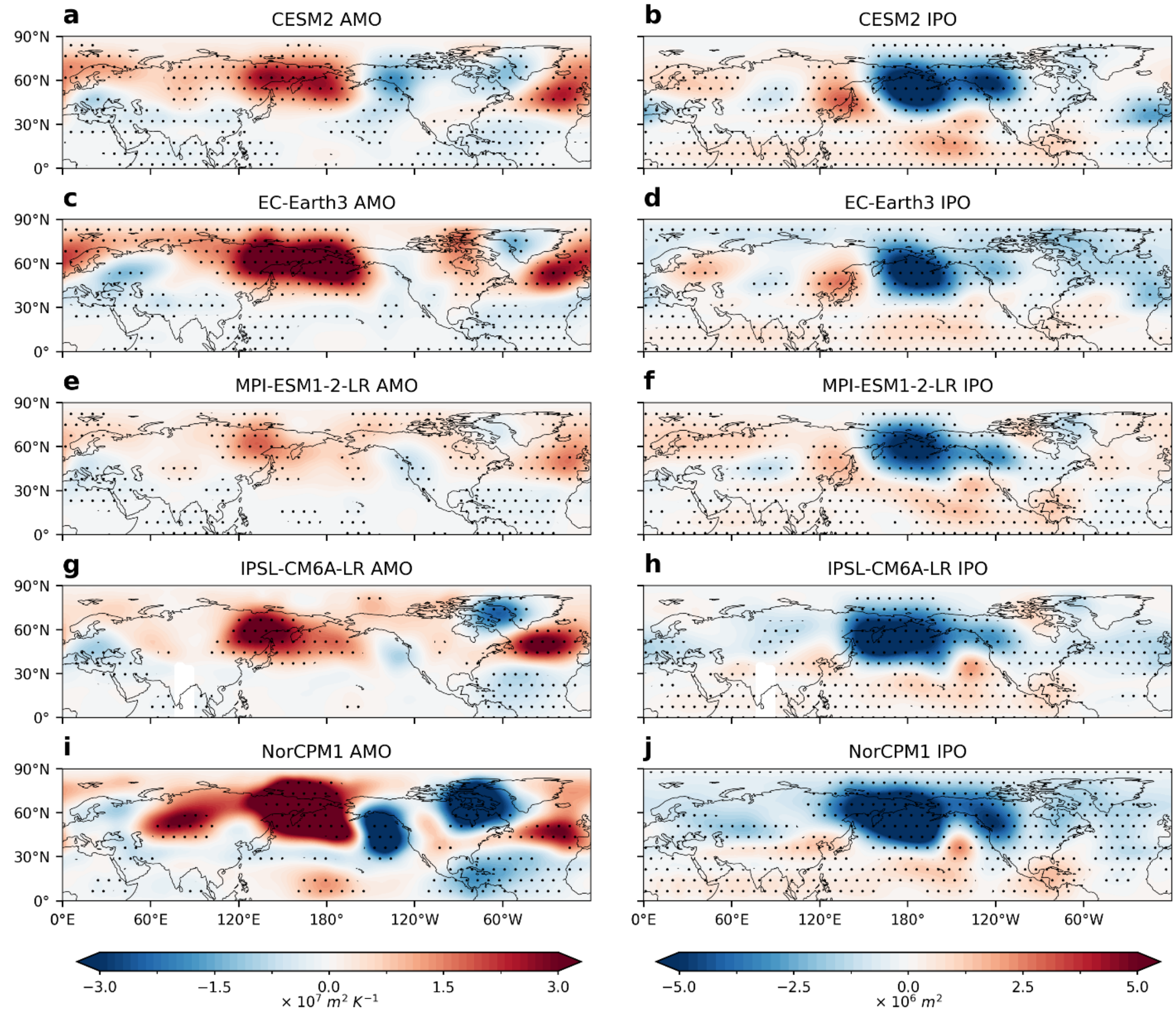

Fig. S12. LWA regression patterns associated with positive Atlantic Multidecadal Oscillation (AMO) and negative Interdecadal Pacific Oscillation (IPO) in five CMIP6 ensembles. **a**, LWA regression pattern associated with positive AMO in CESM2 Large Ensemble (50 members), obtained by regressing LWA variability onto the unstandardized AMO index. **b**, LWA regression pattern associated with negative IPO in CESM2 Large Ensemble, obtained by regressing LWA variability onto the standardized IPO index and reversing the sign so that the pattern corresponds to the negative IPO phase. **c**, **e**, **g**, and **i** are the same as **a**, but for the EC-Earth3 ensemble (50 members), MPI-ESM1-2-LR ensemble (50 members), IPSL-CM6A-LR ensemble (33 members), and NorCPM1 ensemble (30 members), respectively. **d**, **f**, **h**, and **j** are the same as **b**, but for the EC-Earth3 ensemble (50 members), MPI-ESM1-2-LR (50 members), IPSL-CM6A-LR ensemble (33 members) and NorCPM1 ensemble (30 members), respectively. The stippled areas indicate regressions that are significant at the 0.05 level based on the Student's t-test.

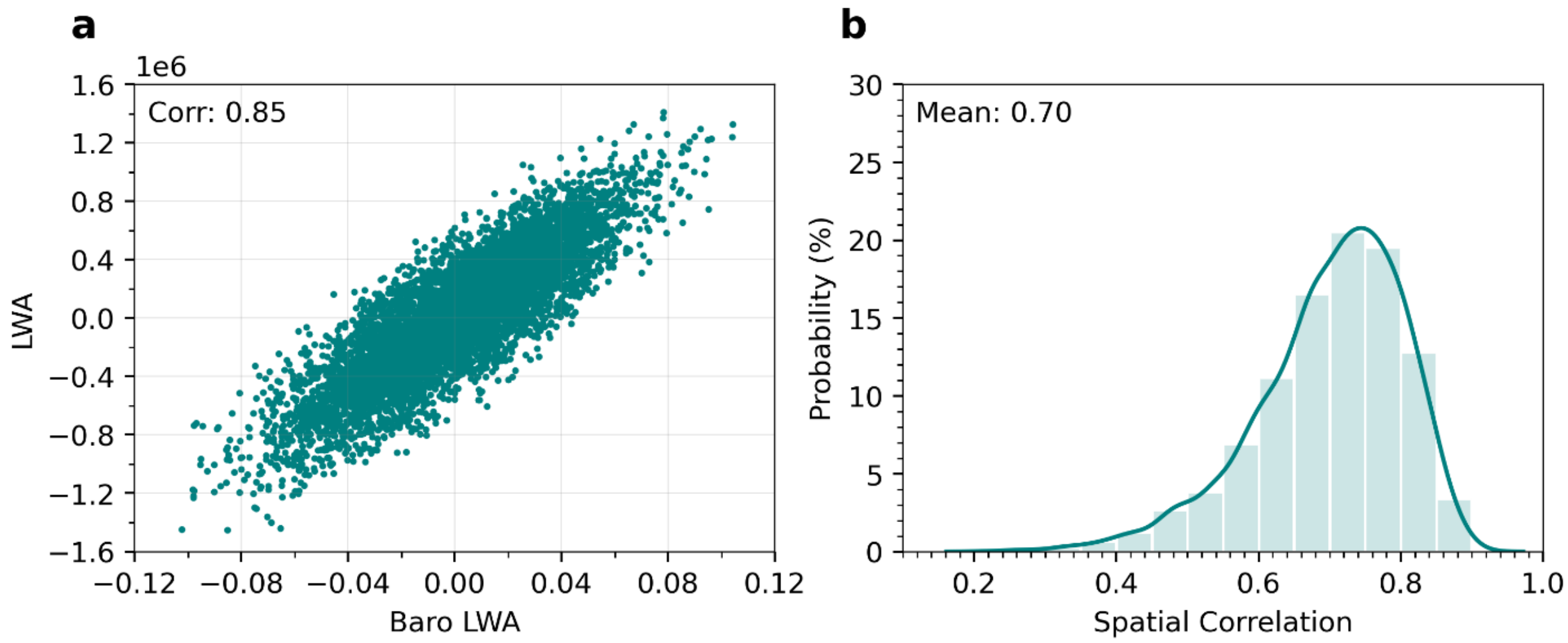

Fig. S13. Correlation between the LWA trends based on 500 mb geopotential height and those based on quasi-geostrophic potential vorticity (QGPV) in CESM2 Large Ensemble. a, Scatter plot between the 23-year LWA trends averaged over the Northern Hemisphere (40N - 80N) based on 500 mb geopotential height and those based on QGPV across the members of the pseudo-ensemble. Their correlation coefficient is shown in the upper left of the panel. **b**, Histograms (bars) and the probability density function based on kernel density estimation (lines) of the spatial correlation between the 23-year LWA trends based on 500 mb geopotential height and those based on QGPV over the Northern Hemisphere (40N- 80N) for the pseudo-ensemble. The mean correlation is shown in the upper left.

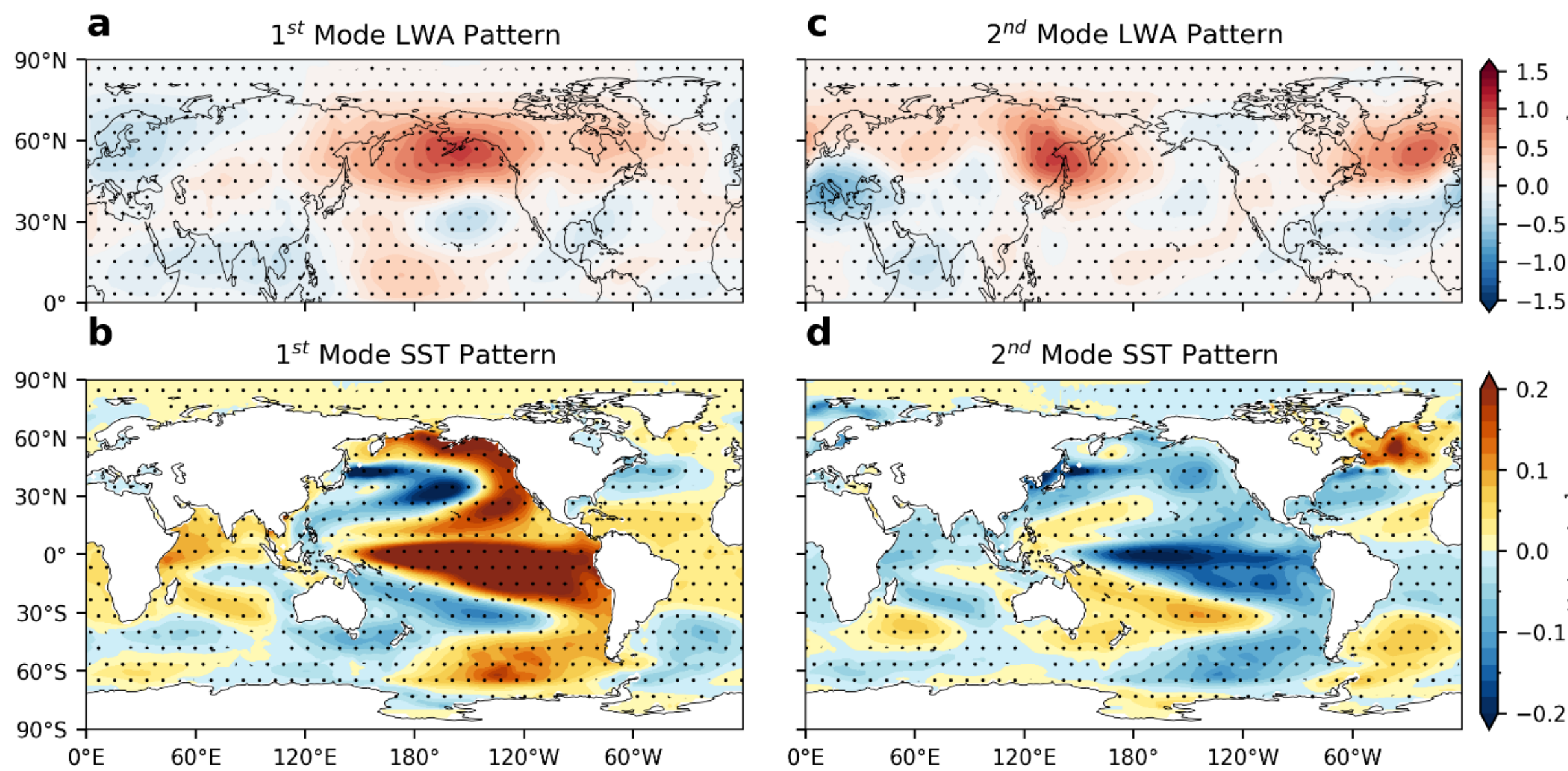

Fig. S14. Leading modes of covarying QGPV-based LWA trends and global sea surface temperature trends due to internal variability in CESM2 Large Ensemble. a, b, Same as Fig. S7a,b, but based on LWA calculated from QGPV. **c, d,** Same as Fig. S8a,b, but based on LWA calculated from QGPV.

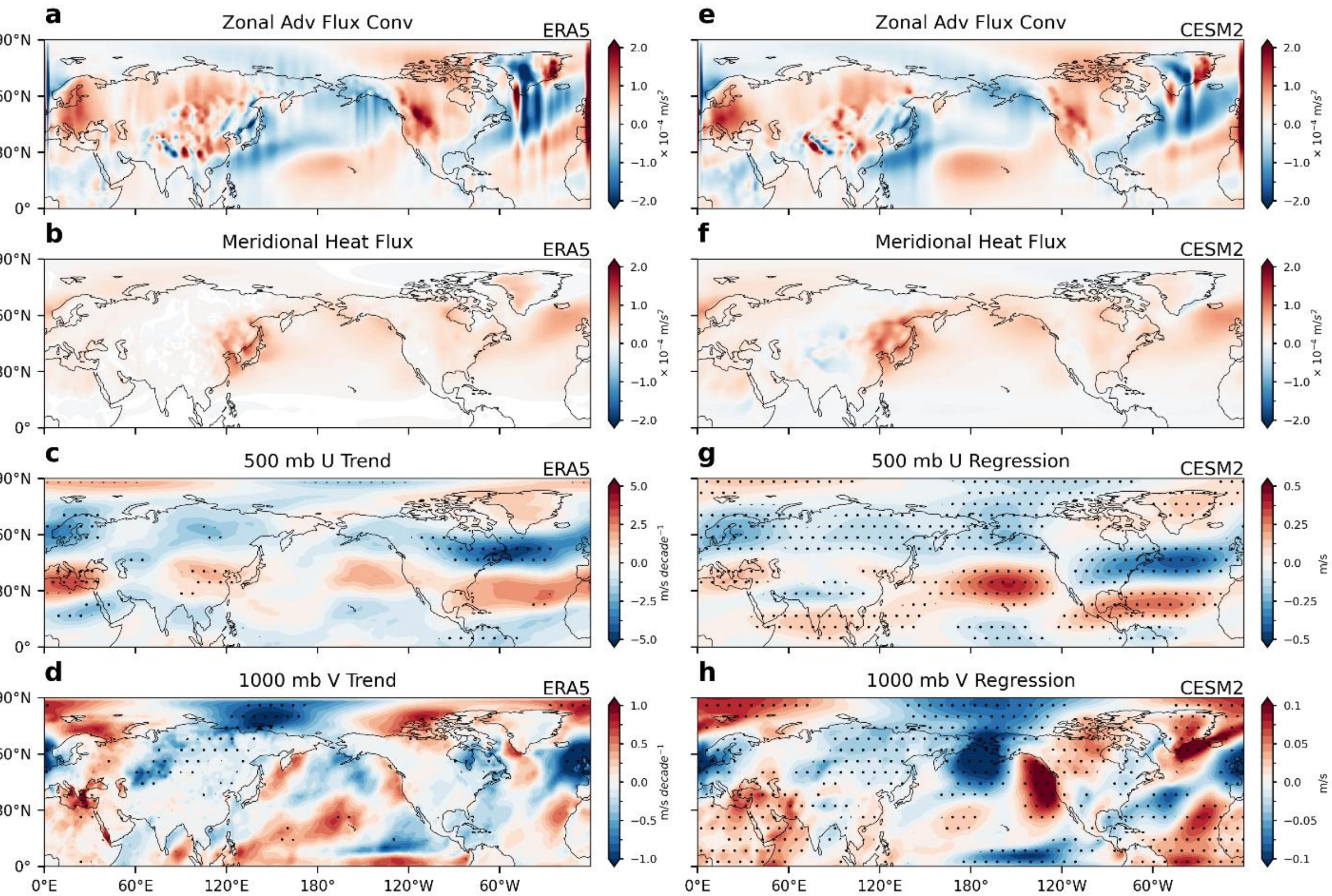

Fig. S15. Mechanisms of changes in zonal advective flux convergence and low-level meridional heat flux associated with positive Atlantic Multidecadal Oscillation (AMO). a, Cold season zonal advective flux convergence climatology in ERA5. **e**, Same as **a**, but for the ensemble mean of CESM2 Large Ensemble. **c**, Cold season low level meridional heat flux climatology in ERA5. **f**, Same as **b**, but for the ensemble mean of CESM2 Large Ensemble. **c**, 500 mb zonal wind trends in ERA5 from 1989-2011. **g**, Regression pattern of 500 mb zonal wind associated with positive AMO. **d**, 1000 mb meridional wind trends in ERA5 from 1989-2011. **h**, Regression pattern of 1000 mb meridional wind associated with positive AMO. The stippled areas indicate trends (**c** and **d**) or regression (**g** and **h**) that are significant at the 0.05 level based on the Student's t-test.

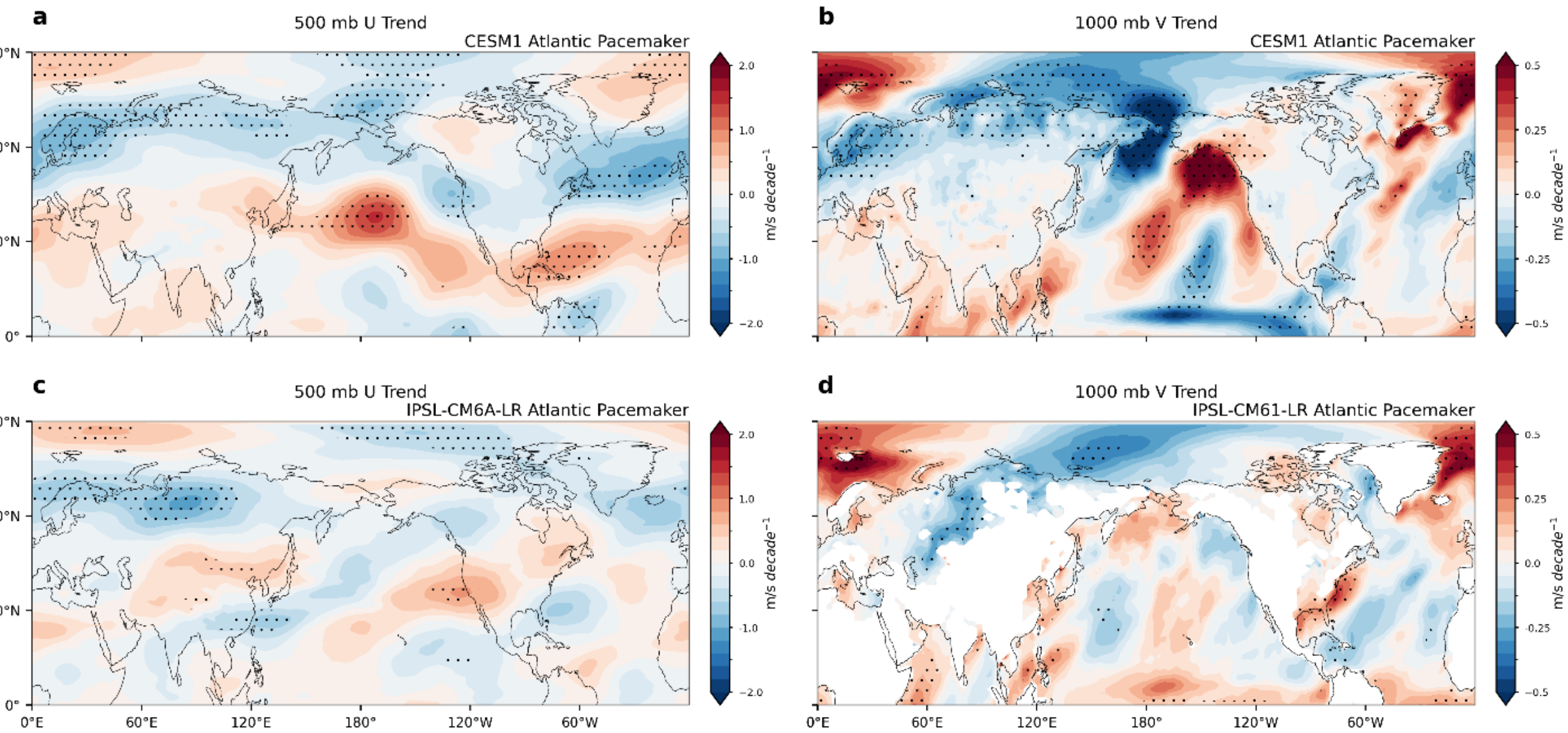

Fig. S16. Trends in 500-mb zonal wind and 1000-mb meridional wind in the Atlantic Pacemaker experiments during DJF 1989–2011. a, Trend in 500-mb zonal wind in CESM1. **b,** Trend in 1000-mb meridional wind in CESM1. **c, d,** Same as **a** and **b**, respectively, but for IPSL-CM6A-LR. Stippling indicates regions where the linear trends are statistically significant at the 0.05 level based on Student's t-test. Note that the color-bar ranges differ from those used in Figs. S15c and S15d.

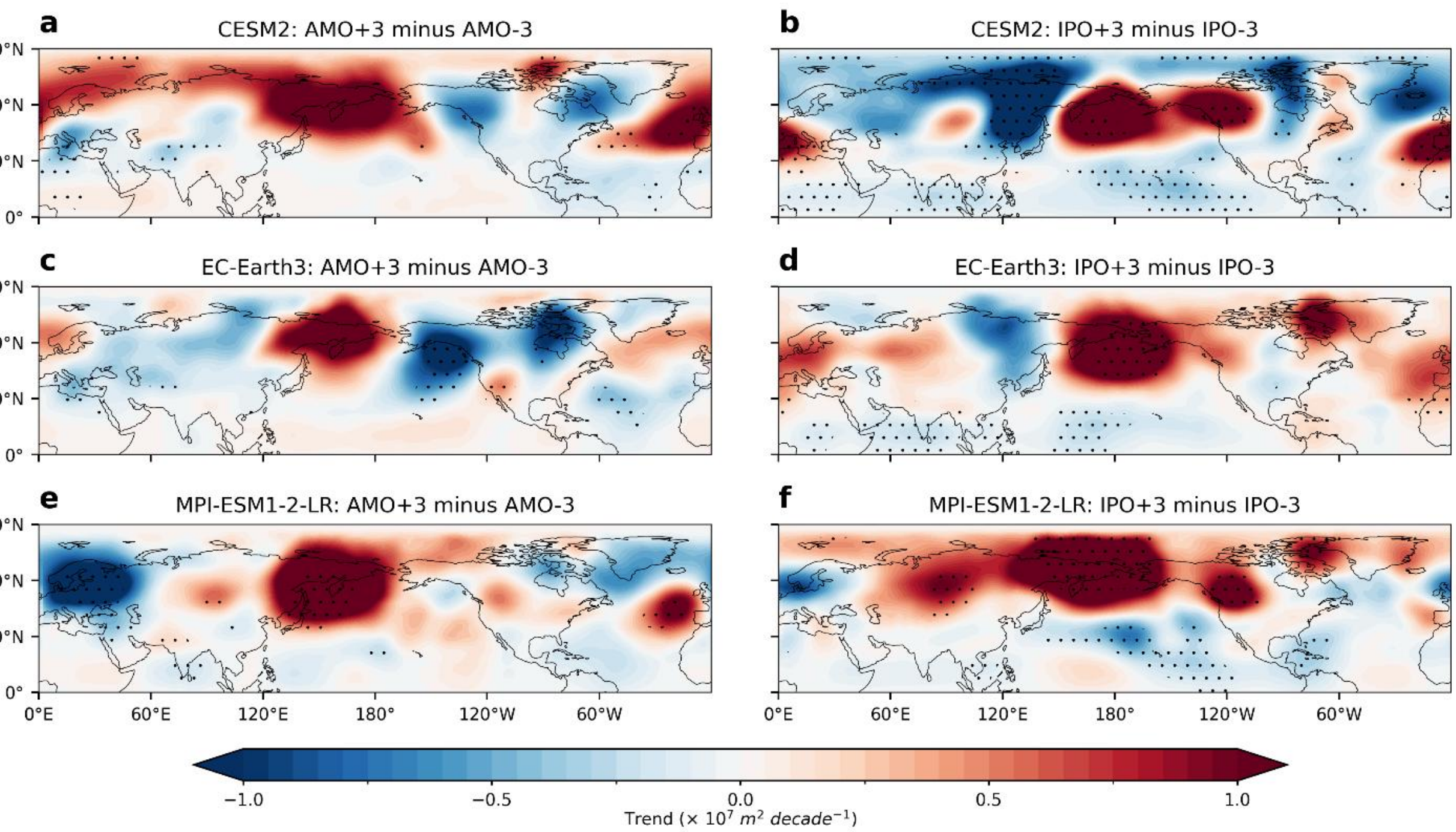

Fig. S17. Near-future LWA trend differences associated with AMO and IPO variability. a, **c**, and **e**, Differences in LWA trends between the three members with the largest AMO trends and the three members with the smallest AMO trends in CESM2 Large Ensemble, EC-Earth3, and MPI-ESM1-2-LR, respectively. **b**, **d**, and **f**, Same as **a**, **c**, and **e**, but for differences between the three members with the largest IPO trends and the three members with the smallest IPO trends. Stippling indicates regions where the trend differences are statistically significant at the 0.05 level based on Student's t-test.

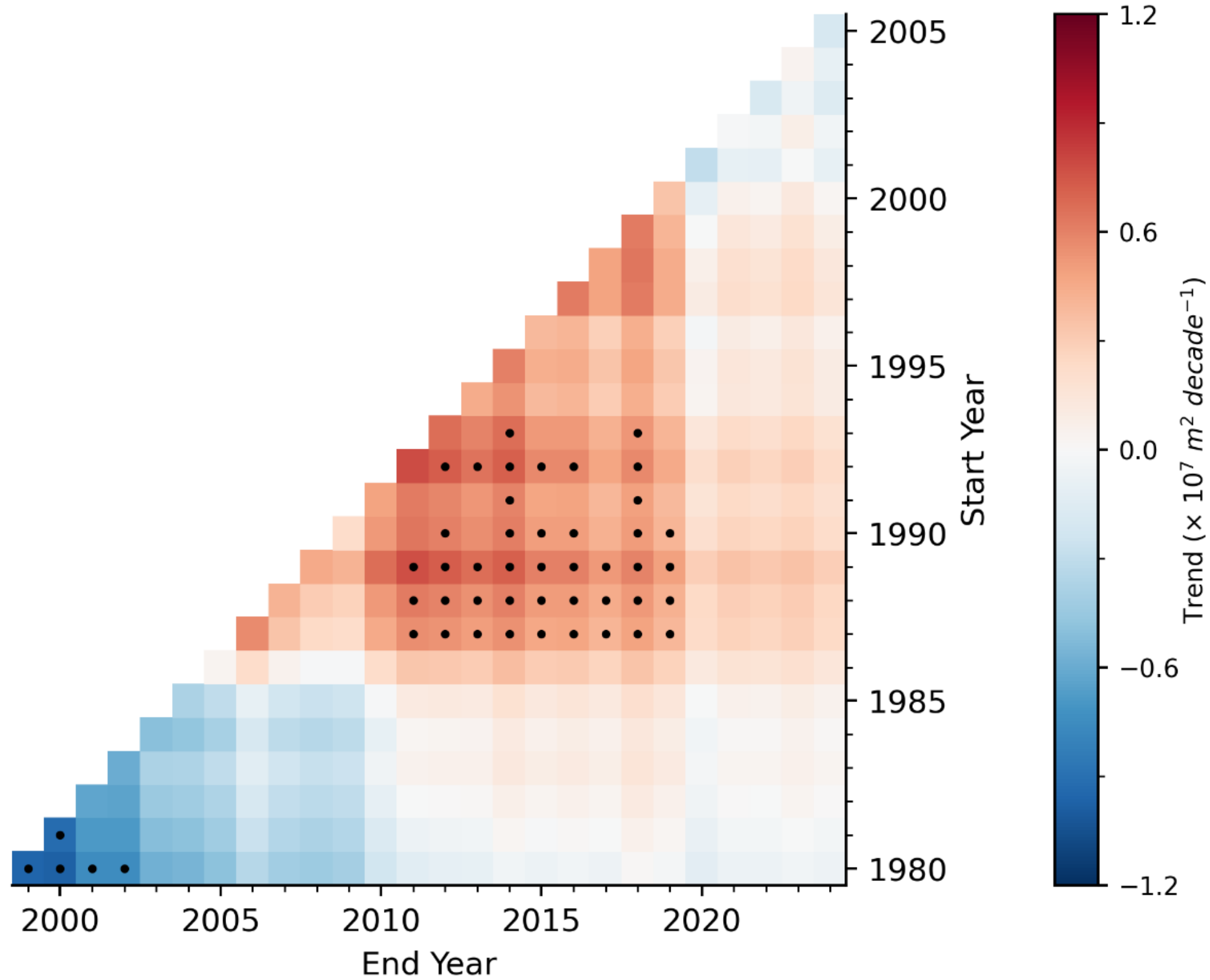

Fig. S18. Observed trends in LWA as a function of start and end year. Observed average LWA trends over the Northern Hemisphere midlatitudes (40°N - 80°N) as a function of start and end year. Only trends of at least 20 years are plotted. The stippling indicates trends that are significant at the 0.05 level based on the Student's *t*-test.
